# Diving on the Surface of a Functional Metal Oxide through a Multiscale Exploration of Drug–Nanocrystal Interactions

**DOI:** 10.1021/acsami.4c19916

**Published:** 2025-02-10

**Authors:** Nicolò Maria Percivalle, Julia Blandine Bassila, Alice Piccinini, Michela Cumerlato, Mariangela Porro, Cheherazade Trouki, Susanna Monti, Giovanni Barcaro, Davide Bochicchio, Roberto Piva, Valeria Rondelli, Giulia Rossi, Valentina Cauda

**Affiliations:** 1 Department of Applied Science and Technology, Politecnico di Torino, Corso Duca degli Abruzzi 24, 10129 Turin, Italy; 2 Department of Physics, Università degli Studi di Genova, Via Dodecaneso 33, 16146 Genoa, Italy; 3 Department of Medical Biotechnology and Translational Medicine, L.I.T.A., Università degli Studi di Milano, V.le F.lli Cervi 93, 20054 Segrate, Italy; 4 Department of Molecular Biotechnology and Health Sciences, University of Turin, Piazza Nizza 44, 10126 Turin, Italy; 5 CNR-IPCF, Institute for Chemical and Physical Processes, Via G. Moruzzi 1, 56124 Pisa, Italy; 6 Department of Pharmacy, University of Pisa, Via Bonanno 6, 56126 Pisa, Italy; 7 CNR-ICCOM, Institute of Chemistry of Organometallic Compounds, Via G. Moruzzi 1, 56124 Pisa, Italy

**Keywords:** drug delivery, zinc oxide, surface functionalization, carfilzomib, small-angle X-ray scattering, molecular dynamics simulations, coarse grained models

## Abstract

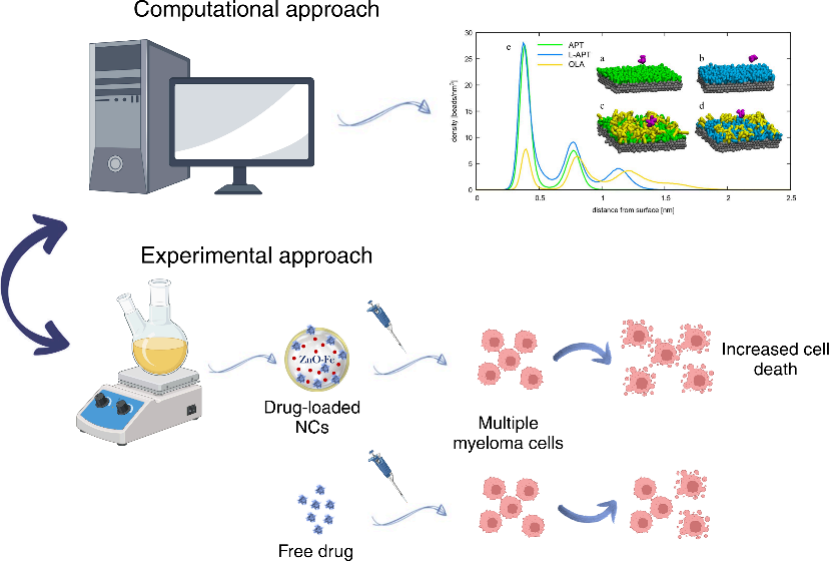

While recent advances in nanotechnology offer significant possibilities for improving the development of targeted drug delivery systems (DDSs), the design of efficient nanocarriers remains challenging due to the complex interactions among nanoparticles, their surfaces, and therapeutic agents in biological environments. To shed light on such difficulties and provide an instrumental tool for the refinement of DDSs, this study presents a comprehensive computational and experimental approach for the development of zinc oxide nanocrystals (ZnO NCs), exploited as carriers for a hydrophobic drug used in the treatment of multiple myeloma (MM), namely, carfilzomib (CFZ). Oleic acid was adopted here as a stabilizing agent during the synthesis of iron-doped ZnO NCs, while aminopropyl groups were used as functionalizing moieties to improve drug adsorption. Advanced characterization techniques were employed to investigate the nanostructure and drug-loading properties. Furthermore, molecular modeling was exploited for elucidating the adsorption mechanism and the thermodynamics of the interactions between the drug and the NCs, offering a detailed understanding at the molecular level. These simulations provided predictive insights into possible molecular inactivation mechanisms and strategies to optimize the nanocarrier design, thus enabling tailored adjustments throughout the development process. While biological tests showed that CFZ-loaded ZnO NCs preserved the drug mechanism of action in MM cell lines, the interconnection between simulations and experiments played a central role in predicting and optimizing NCs–drug interactions. This approach demonstrates the potential of computational simulations in minimizing trial-and-error in the nanoconstruct development process, ultimately streamlining the creation and validation of more effective nanoparticle-based drug delivery systems.

## Introduction

Nanotechnology has brought about infinite possibilities in drug delivery, offering tailored solutions for targeted therapies and personalized treatments against different diseases.^1−3^ Despite the intense research development of nanosized therapeutic tools, it is still an open challenge to deal with the complexity of these nanostructures, their surface, and possible functionalization with chemical moieties and drugs from simple water solution to more complex biological environments.^4−6^

Indeed, the nanoparticles (NPs) effectiveness in drug delivery critically depends on the physicochemical characteristics of their surfaces, on the capability to adsorb and retain drug molecules, and on their biological identity and interaction with target cells.^7−9^ When considering drug delivery nanosystems with an inorganic core, although enormous advancements have been made in the field so far, there is presently a scarce understanding and control of the fundamental physical, chemical, and biological processes involved.^10,11^ This gap of knowledge has so far impaired the translation of these research efforts toward in vivo testing and finally to clinical practice.

**Scheme 1:**
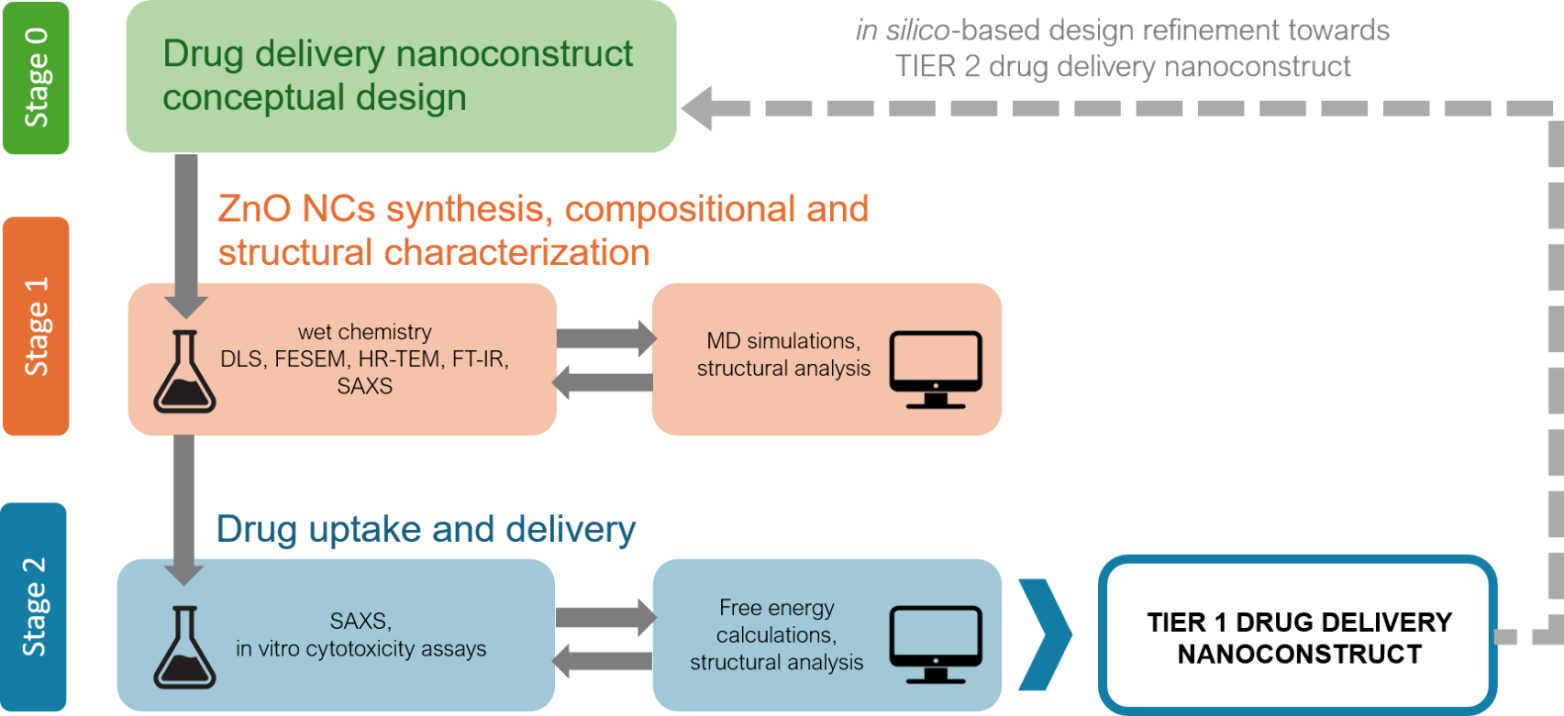
Representation of the Experimental–Computational Approach Followed in This Study, Enabling a Holistic Approach toward the Design and Production of Effective Drug Delivery Nanosystems

In this paper, we propose a multidisciplinary approach consisting of the combination of cutting-edge molecular simulation techniques, experimental design, and characterizations that can enable a holistic approach toward the design and production of effective drug delivery nanosystems, exemplified in Scheme 1.

Among the materials explored for this purpose, functionalized metal-oxide nanoparticles have emerged as a particularly promising avenue. In this context, we focus on this category of nanomaterials, particularly on the well-known zinc oxide nanostructures, due to their extensive use in biomedicine and nanomedicine.^12−15^ Our drug-delivery nanoconstruct is designed to adsorb a hydrophobic drug molecule to be delivered to a therapeutic target. The drug we selected is carfilzomib (CFZ), a second-generation proteasome inhibitor used to treat multiple myeloma (MM), a hematological malignancy.^16−18^ Even though the introduction of proteasome inhibitors has significantly extended survival in MM patients, resistance to these drugs and serious side effects, such as cardiovascular toxicity, remain major challenges. While various attempts to overcome drug resistance have been made in recent years,^19,20^ this issue underscores the need for advanced drug-delivery systems that can precisely target tumor cells, enhancing therapeutic efficacy while minimizing toxicity. We employed our combined experimental–computational approach along the different steps of the nanoconstruct realization, namely, synthesis, characterization, drug loading, and eventually in vitro testing of delivery performances and functionality, i.e., proteasome inhibition.

The experimental synthesis of the proposed zinc oxide nanocrystals (ZnO NCs) evolves from a simple iron-doped metal oxide structure to a more complex system enriching the NCs surface with stabilizing agents, such as oleic acid, and functionalizing ligands, namely, amine-terminated silane, with the aim to tune their drug adsorption and stabilize the NCs in solutions. A variety of techniques, from the high-resolution transmission and scanning electron microscopies, dynamic light scattering (DLS) to small-angle X-ray scattering (SAXS) allow us to fully characterize in situ and ex situ the NCs, their functional surfaces, and capability to adsorb a model drug, carfilzomib. We show that surface functionalization procedures have consequences on NCs colloidal aggregates structure and dispersion as well as on drug adsorption.

Drug loading takes place at a complex interface, and it involves many different molecular players: the nanoparticle core, its organic functionalizing molecules, the drug, and the solvent. We used molecular simulations based on reactive and non-reactive force fields with different resolutions, to characterize such interface and predict the thermodynamics and the dynamics of the drug adsorption. We show that computer simulations can help in finely understanding the mechanism of drug adsorption and shed light on direct surface–drug interactions. Such fundamental knowledge, which is indeed not straightforward at the experimental level, provides insightful interpretation of the complex, reactive environment at the nanoparticle interface, leading to a mechanistic interpretation of the outcomes of the functionalization steps and drug interactions.

To validate our underlying hypotheses, we conducted in vitro biological tests to assess the effectiveness of CFZ-loaded NCs on two MM cell lines, comparing the effects of the free drug to those of the nanocarried formulation. This experimental approach leverages our understanding of the interactions between the drug, the metal oxide surface, and functional groups with the goal of enhancing drug efficacy in cells.

The highly interdisciplinary studies comprising experiments and molecular simulations show how essential it is to conduct experiments and use simulations to confirm or reject hypotheses to, as a perspective, guide or refine the design and fabrication of an effective drug-delivery nanosystem, with full knowledge over the multifaceted and complex key parameters affecting its preparation process. The proposed strategy helps to develop nanoparticles for selected drug-delivery applications, eliminating time-consuming synthesis and test procedures that do not rely on an understanding of the molecular interactions within the NPs–drug complex.

## Materials and Methods

### A Experimental Methods

#### Synthesis of Fe-Doped ZnO Nanocrystals

For the synthesis of iron-doped, oleate-stabilized zinc oxide nanocrystals (ZnO_OLA NCs), a previously optimized wet chemical process was exploited.^21,22^

More specifically, a 100 mL round-bottom flask was filled with 40 mL of ethanol (99%, Sigma-Aldrich) in which 526 mg of zinc acetate dihydrate (Zn(CH_3_COO)_2_·2H_2_O, ACS Reagent, ≥99.0%, Sigma-Aldrich, Darmstadt, Germany) and 58 mg of ferric nitrate nonahydrate (Fe(NO_3_)_3_·9H_2_O, HiMedia) were dissolved. Then, 1 mL of bidistilled water (obtained from a Direct Q3 system, Millipore, Burlington, MA, USA) was added to the solution, which was heated at 70 °C by slightly immersing the flask in a silicon oil bath. The system was positioned on a stirring plate (VELP Scientifica ARE Hot plate stirrer) at 350 rpm and closed in refluxing conditions with a refrigerating column. Once reached the desired temperature, 140 μL of oleic acid (≥99%, Sigma-Aldrich) was added to the solution. After 10 min, a solution prepared by dissolving 1.044 g of tetramethylammonium hydroxide pentahydrate (TMAH, 98.5%, Sigma-Aldrich) in 10 mL of ethanol and 1.052 mL of bidistilled water was quickly added in the flask. After further 10 min, the reaction was stopped by pouring 40 mL of ice-cold (0–4 °C) ethanol in the solution and putting the flask in an ice bath for 3 min. The obtained NCs suspension was centrifuged at 8000 relative centrifugal force (RCF) for 10 min, before discarding the supernatant and resuspending the nanocrystals in 30 mL of fresh ethanol through an ultrasound bath and performing two more washing steps with the same procedure. To have the NCs in water, an aliquot of NCs in ethanol, corresponding to a mass of 1 mg, was collected and a further centrifugation step was applied. The ethanol was discarded, and the needed amount of water was added to reach the desired final concentration from 1 mg/mL up to 3 mg/mL for small-angle X-ray scattering investigation.

#### Chemical Functionalization of Fe-Doped ZnO Nanocrystals

The synthesized nanocrystals were functionalized with aminopropyl groups. The functionalization process was carried out by taking the volume corresponding to 40 mg of NCs and adding it to the amount of ethanol, already poured in a 25 mL round-bottom flask, required to obtain a 2.5 mg/mL dispersion. The solution was then heated at 70 °C by making the bottom of the flask barely touch the surface of a silicon oil bath, all under moderate stirring, in refluxing conditions, and flowing nitrogen gas to strip out humidity. When the condensation in the flask started, 8.6 μL of (3-aminopropyl)trimethoxysilane (APTMS, Sigma- Aldrich), corresponding to 10 mol %, was added to the dispersion. After 6 h of continuous stirring, the final suspension was collected, centrifuged at 14,000 RCF for 10 min, and the functionalized nanocrystals were resuspended in fresh ethanol. Finally, two more washing steps were performed.

#### Nanocrystals Characterization

##### Electron Microscopies

To assess the morphology and the chemical composition of the synthesized nanocrystals, field emission scanning electron microscopy (FESEM, SUPRA 40 from Zeiss, Oberkochen, Germany) coupled with a detector for energy-dispersive X-ray spectroscopy (EDS, x-act 10 mm^2^ silicon drift detector from Oxford Instruments, Oxford, U.K.) was performed. For the sample preparation, 10 μL of a 100 μg/mL concentrated solution of NCs in water was spotted dropwise on a flat silicon substrate, and the measurements were carried out once the sample was completely dry. Transmission electron microscopy (TEM) measurements were performed exploiting a Thermo Scientific Talos F200X G2 S(TEM) operating at 200 kV. For these analyses, the sample was prepared by putting a single drop of a 10 μg/mL NCs solution in water on a copper holey carbon grid and letting it dry.

##### Small Angle X-ray Scattering (SAXS)

SAXS measurements were performed at the BM26 beamline of the European Synchrotron Radiation Facility (ESRF) in Grenoble, France, and at the Austrian SAXS beamline of the ELETTRA synchrotron facility in Trieste, Italy. NCs samples in water were sonicated for 10 min with an ultrasonication bath, immediately before measurements, to ensure full sample dispersion. For measurements, samples were put in quartz capillaries with a 1.5 mm internal diameter at ELETTRA and in polycarbonate capillaries from Enki with an internal diameter of 1 mm at ESRF. Measurements were performed at 22 (room temperature) and 37 °C (physiological temperature). Samples were measured at 3, 2, and 1.6 mg/mL concentration in water. Empty capillaries and capillaries filled with water have been measured in the same conditions for background subtraction.

At ELETTRA, a Pilatus3 1M detector system was utilized with a sample-to-detector distance of 2.3 m to have a q-range from 1.06 × 10^–1^ to 6 nm^–1^ corresponding to the range of distances 1 to 60 nm in the direct space. At ESRF, a Pilatus 1M detector was utilized with sample-to-detector distances of 2 and 9.5 m to collect intensities in the *q*-range 2.7 × 10^–2^ to 6.1 nm^–1^ corresponding to the range of distances of 1–233 nm, employing an energy of 12 keV.

SAXS data are represented as intensity of radiation scattered at the different angles, that is, as a function of the incident-diffracted wave vector difference *q* = (4π /λ)sin θ, with the diffraction angle 2θ and λ being the X-ray wavelength. SAXS data have been analyzed with the fitting program SaSView,^23^ employing a fractal-core–shell model, described in the Supporting Information, section 3. SAXS measurements on ZnO NCs functionalized with oleic acid and APTMS before (ZnO_OLA_APTMS) and after drug adsorption (ZnO_OLA_APTMS_CFZ) were performed on different preparations. SAXS results have been found to be the same (Figure S.5 of the Supporting Information), indicating a reproducible and reliable synthetic protocol for the ZnO NCs and related functionalizations.

##### Wide Angle X ray Scattering (WAXS)

X-ray diffraction measurements were carried out to examine the crystallinity of the synthesized nanocrystals. For this purpose, 1 mg of NCs was redispersed in 50 μL of fresh ethanol before putting 10 μL at a time on a silicon wafer substrate, allowing the previous layer to evaporate. Once completely dry, the analyses were executed with a Panalytical X’Pert PRO diffractometer in Bragg–Brentano configuration, equipped with a Cu Kα monochromatic radiation (λ = 1.540 59 Å) as X-ray source.

##### Optical and Vibrational Spectrscopy Characterizations

Using a sample prepared in the same way as just described, Fourier transform infrared (FT-IR) spectroscopy in the region 4000–400 cm^–1^ range was performed with a Nicolet 5700 FT-IR spectrometer (Thermo Fisher, Waltham, MA, USA).

Furthermore, the NCs optical properties in the UV–vis region were investigated in transmission mode through a double-beam Varian Cary 5000 UV–vis–NIR spectrophotometer. For these measurements, a quartz cuvette (350 μL volume, 1 mm optical path length) was filled with a 2 mg/mL concentrated solution of NCs in ethanol, and a pure ethanol sample as a baseline curve was analyzed for background subtraction.

##### Hydrodynamic Size

The nanocrystal size and surface charge in colloidal solution were assessed through Dynamic Light Scattering (DLS) and ζ-potential measurements, exploiting a Zetasizer Nano ZS90 (Malvern Panalytical, Malvern, U.K.). For the determination of the hydrodynamic radius through DLS, 100 μg of NCs was redispersed in 1 mL of both ethanol and bidistilled water, while the ζ-potential analyses were only performed in bidistilled water.

#### Carfilzomib Uptake

Carfilzomib (CFZ; Selleckchem) uptake was performed by centrifuging 250 μg of NCs and resuspending it in 500 μL of a 1 mg/mL concentrated drug stock in ethanol. The sample was then put on a stirring plate (200 rpm) for 2 h, after which it was centrifuged again in order to also collect the supernatant to be further analyzed. A negative control of pure ethanol and a positive one consisting of the 1 mg/mL drug stock have also been used for the quantification of the CFZ absorbed on the nanocrystals surface.

#### Cell Culture Conditions

Human multiple myeloma (MM) cell lines AMO-1 and KMS-28BM, were obtained from ATCC (American Type Culture Collection, Manassas, Virginia, USA). Cell lines were maintained in RPMI 1640 medium (EuroClone, Pero, Italy), supplemented with 2 mM l-glutamine, 100 U/mL penicillin, 100 μg/mL streptomycin (Gibco), and 10–20% fetal bovine serum (FBS; Sigma-Aldrich, St. Louis, Missouri, USA), and grown at 37 °C in a humidified atmosphere with 5% CO_2_. 293T cells obtained from DSMZ were cultured under standard conditions (37 °C in a humidified atmosphere, with 5% CO_2_) in DMEM supplemented with 10% FBS.

#### *In Vitro* Model To Assess Proteasome Inhibition Sensitivity of MM Cells

##### Generation of pLX301_Ub-G76V-GFP Vector and Lentiviral Particles

pLX301_Ub-G76V-GFP vector was generated from the pEGFP-N1-Ub-G76V-GFP plasmid, a gift from Nico Dantuma (Addgene no. 11941). More in detail, the Ub-G76V-GFP vector was isolated as a single colony and sequenced by Sanger sequencing. The Ub-G76V-GFP casette was ligated into pENTR1A no ccDB vector (Addgene no. 17398) after *Eco*RI and NotI digestion, using T4 ligase. Lentiviral expression vector pLX301_Ub-G76V-GFP was generated by Gateway recombination (Gateway System, Invitrogen) and transformed into DH5α competent cells. Positive clones were confirmed by Sanger sequencing. Lentiviral particles were produced in 293T cells by cotransfecting pLX301_Ub-G76V-GFP and packaging vectors (pCMVdR8.74 and VSV-G) with the Effectene Transfection Reagent (Qiagen), according to the manufacturer’s instructions. Supernatants were harvested over 36 to 60 h, filtrated (0.22 μm pore), concentrated by Lenti-X concentrator (ClonTech), resuspended in cold phosphate-buffered saline (PBS), and stored at −80 °C.

##### Generation of Ub-G76V-GFP MM Cell Lines

pLX301_Ub-G76V-GFP lentiviral particles (10 μL) plus 8 μg/mL Polybrene were used to transduce AMO-1 and KMS-28BM cells (1 × 10^5^/mL). Fresh medium was supplemented 2 h after infection. Stable cell lines expressing the indicated construct were selected by treatment with 1 μg/mL puromycin (Sigma-Aldrich) 24 h postinfection and expanded to completely recover. Cell’s viability was detected by tetrametylrodamine methyl ester (TMRM; Molecular Probes, Eugene, Oregon, USA) staining-flow cytometry 72 h and 10 days postinfection.

##### Assessment of Ub-G76V-GFP Modulation in MM Cell Lines

To assess the proteasome inhibition sensitivity of the Ub-G76V-GFP system, the mean fluorescence of the GFP+ cells was measured by flow cytometry. In detail, Ub-G76V-GFP MM cells were seeded at 1 × 10^5^ cells/mL in 24 well-plates and treated with increasing concentrations (0–1.25 nM–2.5 nM and 5 nM) of CFZ. The rationale for using 1.25, 2.5, and 5 nM concentrations of free CFZ in MM cell testing is based on the IC_50_ determined in our previous work.^24^ Briefly, 1.25 and 2.5 nM represent sublethal concentrations of CFZ, while 5 nM corresponds to the IC_50_ in the AMO-1 cell line. Cell viability, percentage of GFP+ cells, and GFP+ mean fluorescence were measured at 6, 12, and 24 h post-treatment by flow cytometry.

##### Assessment of Drug Loading on NCs Surface through MM Cells Viability Monitoring

The cytotoxic effect of drug-loaded NCs was tested on both the AMO-1 and KMS-28BM cell lines. To this aim, the carfilzomib loading on the ZnO NCs was carried out as above, the supernatant was removed, and the nanocrystals were resuspended in double distilled water at a concentration of 10 mg/mL. Finally, both cell lines were treated with a concentration of 5 and 10 μg of drug-loaded NCs/mL of medium, and their viability was assessed after 24 h. As a control, the same amount of nanocrystals without a drug loaded on their surface was administered to the cells.

A second experiment was conducted with carfilzomib-loaded NCs to further evaluate their cytotoxicity at 24 h. In this case, once the drug loading process was completed, 5 and 10 μL (per mL of cell culture medium) of the postuptake supernatant was directly administered to both cell lines, before performing three additional washing steps (14000*g*, 10 min) in cell culture medium. After each washing, 5 and 10 μL of the resulting supernatants was again administered to different populations of both AMO-1 and KMS-28BM cell lines. A further set of both cell lines was treated with the washed NCs at concentrations of 5 and 10 μg of NCs/mL of medium.

For both experiments, cell viability was assessed by adding 10 μL of WST-1 (Cell Proliferation Reagent WST-1, Roche) to each well. After 2 h of incubation at standard conditions, the formazan absorbance was detected at 450 nm through the Multiskan Go microplate spectrophotometer (Thermo Fisher Scientific) using a 620 nm reference.

A final experiment was conducted on the engineered Ub-G76V-GFP AMO-1 cell line to assess the bioactivity of the CFZ-loaded NCs. Also in this case, cells were seeded at 1 × 10^5^ cells/mL in 24 well-plates and treated with increasing concentrations (0–1.25 nM–2.5 nM and 5 nM) of CFZ, as well as CFZ-loaded NCs at concentrations of 5 and 10 μg of NCs/mL of medium washed three times. Cell viability, percentage of GFP+ cells, and GFP+ mean fluorescence were measured at 12, 24, and 48h post treatment by flow cytometry.

#### Statistical Analyses

Statistical analyses were performed with GraphPad Prism 10.0 (GraphPad Software Inc., San Diego, CA, USA). Statistical significance of differences observed was determined by two-way/two-way ANOVA; differences were considered significant when the *p*-value was <0.05 (*), <0.01 (**), <0.001 (***), or <0.0001 (****).

### B Computational Methods

#### Preliminary All-Atom Molecular Dynamics Simulations Used To Prepare the CFZ Coarse-Grained Model

First, to identify the most probable conformations of CFZ, we used the Balloon program (version 1.6.4),^25,26^ which iteratively changes torsion angles, double bonds, chiral centers, and ring structures through a multiobjective genetic algorithm and then energy-minimizes and selects the structures through a modified version of the MMFF94 force field.^27^ The initial geometry of CFZ was downloaded from the RCSB Protein Data Bank (RCSB PDB) (code: 4R67), and then the program was run, choosing 200 generations with an initial population of 50 conformers. We fixed the ring structures and trans-peptide bonds. Thus, we explored ten torsional angles of the backbone and nine dihedrals of the side chains using the electronegativity equalization method (EEM) as an atomic charge model. The program produced 356 conformers, which were reduced to 126 structures by excluding similar backbone arrangements. These structures were favored by intramolecular interactions between neighboring residues (i.e., hydrogen bonds of the backbones and stacking/T-shaped orientations of the rings). These were reoptimized at the quantum chemistry (Q.C.) M06-2X/6-31G(d) level in the gas phase with the Gaussian09 code (revision A02).^28^ All the conformations were analyzed and classified in terms of torsional angles, head–tail and ring–ring distances, number/type of intramolecular hydrogen bonds, and secondary structure characteristics.^29^

To characterize the structure variability of CFZ in water solutions at ambient temperature, we carried out classical molecular dynamics (MD) simulations with the AMBER16 package,^30^ using the GAFF2 force field and the TIP3P explicit water model with RESP charges (calculated at the DFT/M06-2X/6-31G(d) level of theory). Minimum and maximum CFZ energy structures were selected as starting geometries of the M.D.s and separately simulated after insertion in a 13812 water box (76.9 × 71.8 × 73.5 Å^3^).

The initial equilibration consisted of *NVT* and then *NPT* simulations for about 600 ps at constant temperature (300 K), using Andersen’s thermostat/barostat with isotropic molecule-based scaling and a time constant of 1 ps. All Lennard-Jones interactions were cut off at 12 Å, and a particle mesh Ewald correction for the long-range electrostatic contribution was applied. The integration step was set to 1 fs. Production simulations in the *NVE* ensemble were carried out for about 50 ns, with the configurations saved every 0.1 ps. The analysis was focused on dihedral angle, ring–ring distance, head–tail distance, and radius of gyration distributions (all these data are shown in the Figures S.1 and S.2 of the Supporting Information).

#### Molecular Dynamics Simulations at the Coarse-Grained Level and the Related Models

Coarse-grained simulations were performed using the Martini 3 force field (Souza et al., 2021). The Martini model employs beads made of groups of atoms classified according to their hydrophobicity. It includes apolar (C), nonpolar (N), polar (P), and charged (Q) beads. Depending on the number of non-hydrogen atoms represented by a coarse-grained bead, they can have three different sizes, namely, tiny (2 atoms), small (3 atoms), and regular (4 atoms). Further details on the Martini force field model can be found in literature.^31,32^ Martini 3 models were developed for all the materials and molecules involved in this work, namely, carfilzomib, zinc oxide, oleic acid, APTMS, and N-(2-Aminoethyl)-3-aminopropyltrimethoxysilane (L-APTMS), as shown in Figure S.3 of the Supporting Information.

#### Coarse-Grained Molecular Dynamics Simulation Setup

Gromacs software, version 2020.6, was exploited to perform simulations and analyze trajectories. The *md* integrator algorithm was used to integrate Newton’s equations of motion with a time step of 20 fs. The simulation time was 5 μs. The neighbor list was updated every 20 steps with a buffer tolerance of 0.005 kJ mol^–1^ ps^–1^. Periodic boundary conditions were employed in all directions. The reaction-field scheme was exploited to treat electrostatic interactions with a cutoff of 1.1 nm and a relative dielectric constant of 15, and a cutoff of 1.1 nm was used for van der Waals interactions. Temperature was maintained at 310 K with the *v-rescale* heat bath,^33^ with a time constant for coupling of 1 ps. In all systems, a pressure of 1 bar was applied only in the *z* direction of the simulation box with the semi-isotropic Parrinello–Rahman pressure coupling.^34^ The time constant was set at 12 ps and the compressibility to 3 × 10^–4^ bar^–1^. Along the *x* and *y* directions, no pressure coupling was applied. Constraints in the phenylalanine ring structure of carfilzomib were treated with the LINCS algorithm.

#### Calculation of the Free Energy Profiles of CFZ Adsorption on the Functionalized Surface

Metadynamics simulations of systems made of one CFZ plus a surface functionalized with two ligands, namely, OLA and APTMS/L-APTMS, were performed. The *z* component of the distance between the solid surface and the center of mass of CFZ was selected as a collective variable to describe the adsorption of CFZ on the surface. A value of 0.1 nm was used for the width of the Gaussian hill, while its height was set to 0.2 kJ/mol. Gaussian functions were added every 5000 steps (corresponding to 0.1 ns), and a wall was used to restrain the collective variable to a maximum value of 4 nm. The force constant (*KAPPA*) for the wall was set at 250 kJ mol^–1^ nm^–2^ and the exponent determining the power law (*EXP*) was set to 2. Once the simulation was completed, the free energy profiles (Figure 5) were obtained by averaging 10 independent free energy profiles at convergence using an in-house python script. The error bars were obtained as standard errors.

## Results and Discussion

The Results section is structured to systematically present the findings derived from our experimental and computational investigations, highlighting their complementary contributions to the study. We will proceed from material synthesis to the biological validation of the nanoconstruct. The section begins with the physicochemical characterization of the synthesized ZnO nanocrystals (NCs), including their morphology, size distribution, and surface functionalization. This is followed by exploration, mainly via SAXS, of the structural and aggregation properties of NCs in solution. Subsequently, the interaction of the drug (CFZ) with the functionalized NCs is predicted and analyzed through a combination of molecular dynamics simulations, SAXS and experimental drug uptake assays. Finally, the biological efficacy of CFZ-loaded NCs is evaluated in multiple myeloma cell lines, emphasizing their potential as a drug delivery system.

### ZnO Nanocrystals Physicochemical Characterization

**Figure 1:**
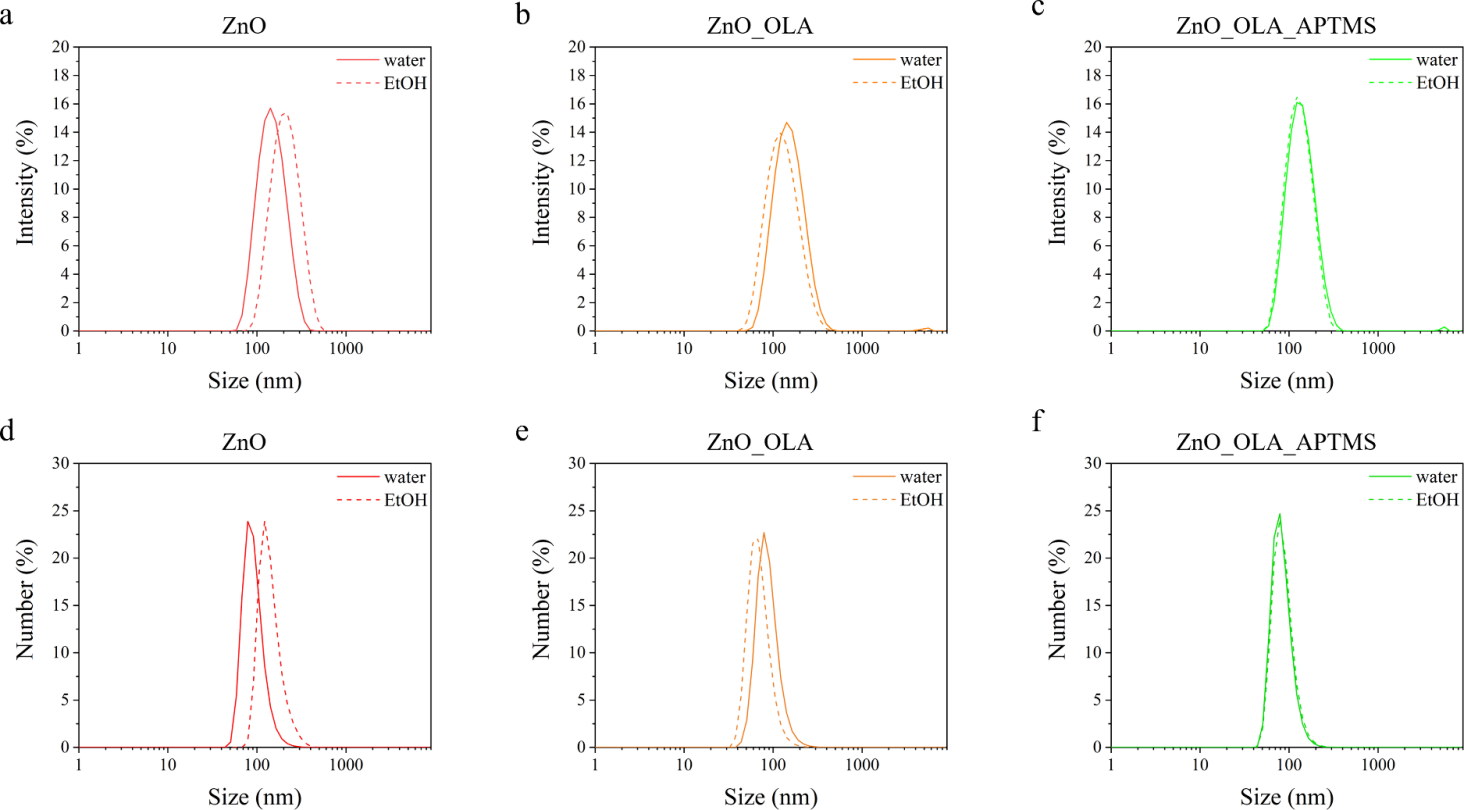
DLS measurements of (a, d) ZnO, (b, e) ZnO_OLA, and (c, f) ZnO_OLA_APTMS NCs in double distilled water and ethanol. The upper row reports the size distributions in % intensity, while the lower row reports the distributions in % number.

We started synthesizing the ZnO nanocrystals, according to the wet-chemistry procedure reported in the Materials and Methods section, and characterizing the colloidal product obtained. DLS measurements (Figure 1) showed a narrow size distribution and monodispersity index for all the analyzed NCs, from the pristine to multifunctionalized surfaces, in both ethanol and water (see also Table 1 for the specific values).

**Table 1:** Average Hydrodynamic Diameters (Expressed as % Number), Polydispersity Index (PDI), and ζ-Potential Values of Iron-Doped ZnO, ZnO_OLA, and ZnO_OLA_APTMS Nanocrystals in Ethanol and Double Distilled Water

sample	average hydrodynamic size (nm)	PDI	ζ-potential (mV)
ZnO NCs EtOH	122.1	0.12	na
ZnO NCs water	79.5	0.11	+28.5
ZnO_OLA NCs EtOH	67.8	0.15	na
ZnO_OLA NCs water	79.7	0.16	+27.6
ZnO_OLA_APTMS NCs EtOH	78.8	0.15	na
ZnO_OLA_APTMS NCs water	78.8	0.12	+34.8

The narrow and uniform size distribution of the analyzed samples is also supported by the high values of the ζ-potential of the NCs in water, as reported in Table 1. In particular, the highest ζ-potential value is obtained for the ZnO_OLA_APTMS NCs and is equal to +34.8 mV. This result, paired with the low values of the PDI, reasserts the good colloidal stability of the nanocrystals functionalized with APTMS in combination with the oleic acid capping. Further stability tests were performed on ZnO_OLA_APTMS NCs in RPMI 1640 cell culture medium alone and complemented with 10% FBS. The results of these measurements are reported in the Supporting Information (Figure S.4).

**Figure 2:**
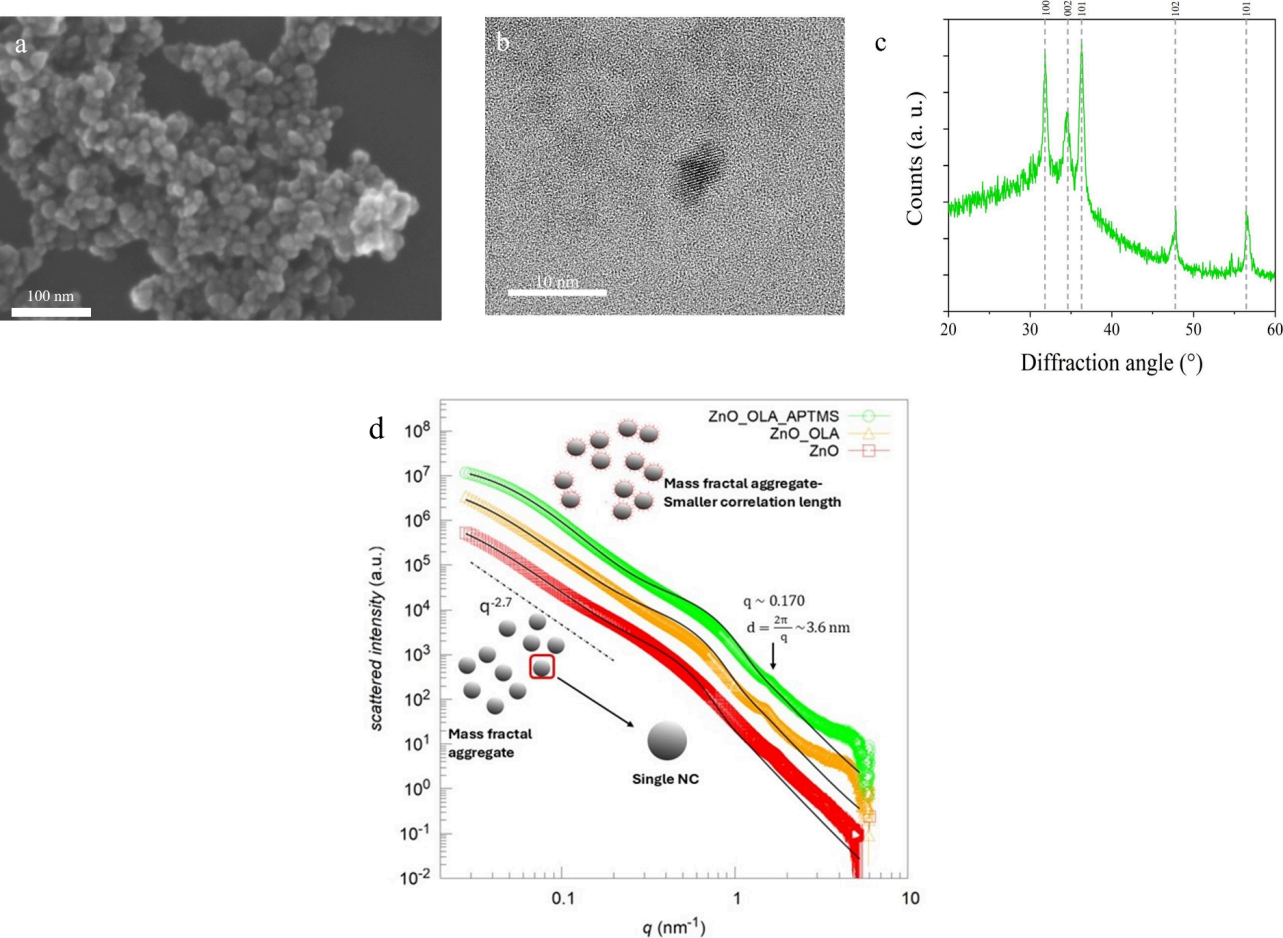
(a) Field emission scanning electron microscopy (FESEM) image of the synthesized ZnO_OLA_APTMS NCs; (b) high-resolution transmission electron microscopy (HR-TEM) image of ZnO_OLA_APTMS NCs; (c) X-ray diffraction pattern of the analyzed ZnO_OLA_APTMS NCs; (d) SAXS profiles (symbols) of ZnO nanocrystals without and with functionalizations together with their best fits (black lines). Red squares, ZnO-Fe NCs; orange triangles, ZnO_oleic acid (ZnO_OLA); green circles, ZnO_OLA_APTMS. ZnO_OLA_APTMS and ZnO_OLA_APTMS_CFZ are represented with an incremental multiplication factor of 5 for better visibility.

The complete iron-doped ZnO NCs, capped with oleic acid and functionalized with APTMS, were further characterized by FESEM analyses (Figure 2a) highlighting the successful wet-chemical synthesis resulting in round-shaped nanocrystals, with a diameter of around 5–8 nm. The size of the nanocrystals was then confirmed by the results derived from the HR-TEM measurements (Figure 2b).

By comparison of the DLS results with the electron microscopy characterizations, it is clear that the NCs tend to aggregate in uniform aggregates below 100 nm in both ethanol and water. It is worth noting that the electron microscopy measurements are performed on dry samples under ultrahigh vacuum, while for DLS analysis the NCs are suspended in solution and an average hydrodynamic diameter is evaluated. Higher resolution techniques to unravel these functionalized NCs structures as colloidal suspensions are indeed needed, and SAXS can offer a broad insight into such details, as demonstrated below.

Besides the size validation, HR-TEM analysis provided more in-depth information about the structure of the NCs, being a single-crystalline structure and attributed to the typical wurtzitic arrangement of ZnO, as assessed through XRD measurements (Figure 2c). The characteristic diffraction peaks of the ZnO wurtzitic structure were indexed and reported in Table S.1. Further discussion on the crystalline structure and iron doping is provided below on SAXS data.

The chemical composition of the synthesized nanocrystals was also analyzed by FT-IR measurements (Figure S.5a in the Supporting Information). All the surface functional groups, like APTMS and OLA are present, as expected. The semiconductor properties of ZnO are also well confirmed (Figure S.5b of the Supporting Information), with the optical band gap was calculated through the Tauc’s plot exploiting the direct semiconductor equation, resulting equal to 3.4 eV.

### Characterization of the ZnO NCs and the Different Functionalizations by Small Angle X-ray Scattering

The structure and the *in-solution* behavior of the NCs were studied with SAXS, as it is indeed an ideal technique to determine the shape, size, and structure of iron-doped ZnO NCs, due to high electronic density of ZnO and Fe atoms.

The pristine iron-doped ZnO NCs were first studied in liquid media, and then the samples with the different functionalizations (OLA and then APTMS) were studied progressively, to understand the role of each functionalization on NCs structure (Figure 2d). The SAXS spectra show, in the intermediate q-vector range, the features characteristic of small (tenth of nm typical distances in the direct space) particles. At low q-vector, the spectra show a slope with a power law decay that is associated with the large distance scale (10–100 nm) structuring of the systems. It is thus possible to extrapolate information on the mass fractal features. In high-q region (relative to a few nm typical distances in the direct space) the difference in intensity between the experimental data and the fit indicates that small-sized objects with high electron density are present in solution. The model applied for data fitting, described in detail in the Supporting Information, section 3, accounts for the presence of spherical particles in solution, possibly with a core–shell structure, aggregated in a disordered arrangement of finite size with fractal dimensionality (from the low q-vector SAXS profiles slopes), related to the compactness/looseness of the scattering structures of a self-similar nature. The fractal dimension of the different particle systems was found to be between −2.6 and −2.9 (Table S.2 of the Supporting Information), depending on the NC’s functionalization. This interval corresponds to the mass fractal dimensionality range, where the lowest fractal dimension was found for the iron-doped ZnO sample and increases at increasing functionalization levels. The higher the fractal dimension, the more the particles are correlated. This indicates that functionalizations allow NCs to be more compact in solution. Meanwhile, the correlation length, which is related to the size of homogeneous areas in the samples, related to clusters size, was found to decrease at increasing the number of functionalizations. From the intermediate q-vector region of SAXS profiles, we can have information about the size of the clustered spheres, between 4.6 and 6.6 nm, with a polydispersity of around 40%.

In summary, SAXS data at low and intermediate q-vector values indicate that at increasing functionalizations of the ZnO surface, the system compactness is higher and the size of homogeneous areas, i.e. NCs aggregates in water solution (see Table 2). This last finding can be directly linked to the decrease in NCs size in solution upon functionalization, as observed by DLS in Figure 1. SAXS data, indeed, corroborate the hypothesis raised by DLS and FESEM/HR-TEM comparison, that the iron-doped ZnO NCs are composed of small core particles sized around 6 nm, clustering in bigger aggregates in solution. This result highlights the positive role of functional moieties in providing a better dispersion in water of the NCs. Given the very high electron density of iron-doped ZnO, more complex features such as the presence of surrounding functional shells of different moieties as oleic acid or APTMS, cannot be ascertained. However, the presence of such surrounding shell cannot be excluded as it is still consistent with the measured profiles. We thus performed an analysis adding a shell to the core of the fractal model and fixed the scattering length densities (SLDs) of the different NCs without and with functional moieties to the values reported in Table S.3 of the Supporting Information. We can observe that, in the case of oleic acid, a thin shell with low coverage can be formed around the metal oxide core. In the presence of both oleic acid and APTMS, the shell thickness is not affected while the shell coverage on the ZnO core reaches the value of 100% (details in Table 2). Previous investigations from Bauer et al. indicate indeed the role of APTMS in making a more uniform and well-formed coating shell surrounding ZnO NCs.^35^ Our data of NCs functionalized with APTMS are compatibly fitted with the presence of a uniform hydrophobic layer (formed by APTMS and OLA) around the metallic core.

**Table 2:** Fit parameters of SAXS Data Using a Model of Fractal-Core Shell, by SasView^a^

	radius (nm)	Th_sh_ (nm)	fractal dimension	correlation length (nm)	Bragg spacing (nm)	SLD shell (10^–6^ Å^–2^)
ZnO-Fe	3.3	0	2.6	39	3.6	—
ZnO_OLA	2.8	0.6	2.7	33	4.1	9.2
ZnO_OLA_APTMS	2.3	0.6	2.9	20	3.9	8.5

a Radius (inner core), Th _sh_ = thickness of the shell, SLD _sh_ = SLD of the shell, Bragg spacing = 2π/*q* from peak position (∼1.7 nm^–1^). Scattering length density (SLD) of the core was fixed at 45 × 10^–6^ Å^–2^. The polydispersity of the radius is assimilated to the width of the distribution of ±40%. The uncertainty of the fitting parameters for shell thickness was estimated at ±0.1 nm, for SLD _sh_ at 0.2 × 10^–6^ Å ^–2^, while for the correlation length ±10%.

Fits depart from experimental data in the highest *q*-range region, suggesting that small particles (1 nm sized) with high electron density may be present in the solution, possibly being tiny metal oxide NCs. Also, the departure of the bare ZnO spectrum slope at high *q* from −4 indicates that pristine NCs have a rough surface.

In addition, a Bragg peak at *q* ∼ 1.72 nm ^–1^, relative to a distance in the direct space of 3.6 nm, can be distinguished in all the SAXS spectra. Previous SAXS studies on ZnO materials reported on the presence of Bragg peaks, at approximately the same q-vector;^36^ authors referred to this peak as being associated with domains of high-contrast dopers inside ZnO NCs. Similarly, in our samples, a strong contrast may be offered by the iron doping element, which might partition, for example, just in ZnO NCs external layers or just in the NCs core, giving then rise to a characteristic repeating distance into the sample. Also, such peaks can derive from piled homogeneous iron-doped ZnO NCs; considering the dimension of the NCs, indeed, this peak can be associated with the distances among ZnO NCs, possibly from facing planar faces, forming a 5–8 nm aggregate in size populating the fractal. The functionalizations of ZnO NCs with oleic acid and APTMS induce a shift of this peak toward smaller *q* values, i.e., toward bigger distances among the subunits forming the 5–8 nm NCs (see Table 2).

At varying NCs concentrations in solution from 1.6. to 3.6 mg/mL, none of the observed features were found to be modified, indicating that NCs structure is maintained.

### Study of Drug Uptake: All-Atom and Coarse-Grained Molecular Dynamics Simulations

Prior to performing the experimental adsorption of the highly hydrophobic CFZ drug on the functionalized and iron-doped ZnO NCs surface, molecular dynamics simulations were conducted. To finely explore the behavior of the drug at the interface between the NCs and the environment, we employed both all-atom and coarse-grained M.D. simulations. All-atom simulations were basically used to prepare a richer database of CFZ structures compared to the one already produced in a previous study,^29^ to obtain a more reliable parametrization of the coarse-grained model. Notwithstanding the extension of the sampled time to 50 ns, the analysis of the data showed a consistent trend of the structural organization of the backbone and side chains of the molecule in water solution, confirming the great flexibility of CFZ and its tendency to adopt both elongated and coiled configurations characterized by intramolecular hydrogen bonds and T-shaped/stacked ring-side arrangements (Figures S.1 and S.2 of the Supporting Information). The development of the coarse-grained model was performed as described in the Supporting Information (Figure S.3 and related text).

**Figure 3:**
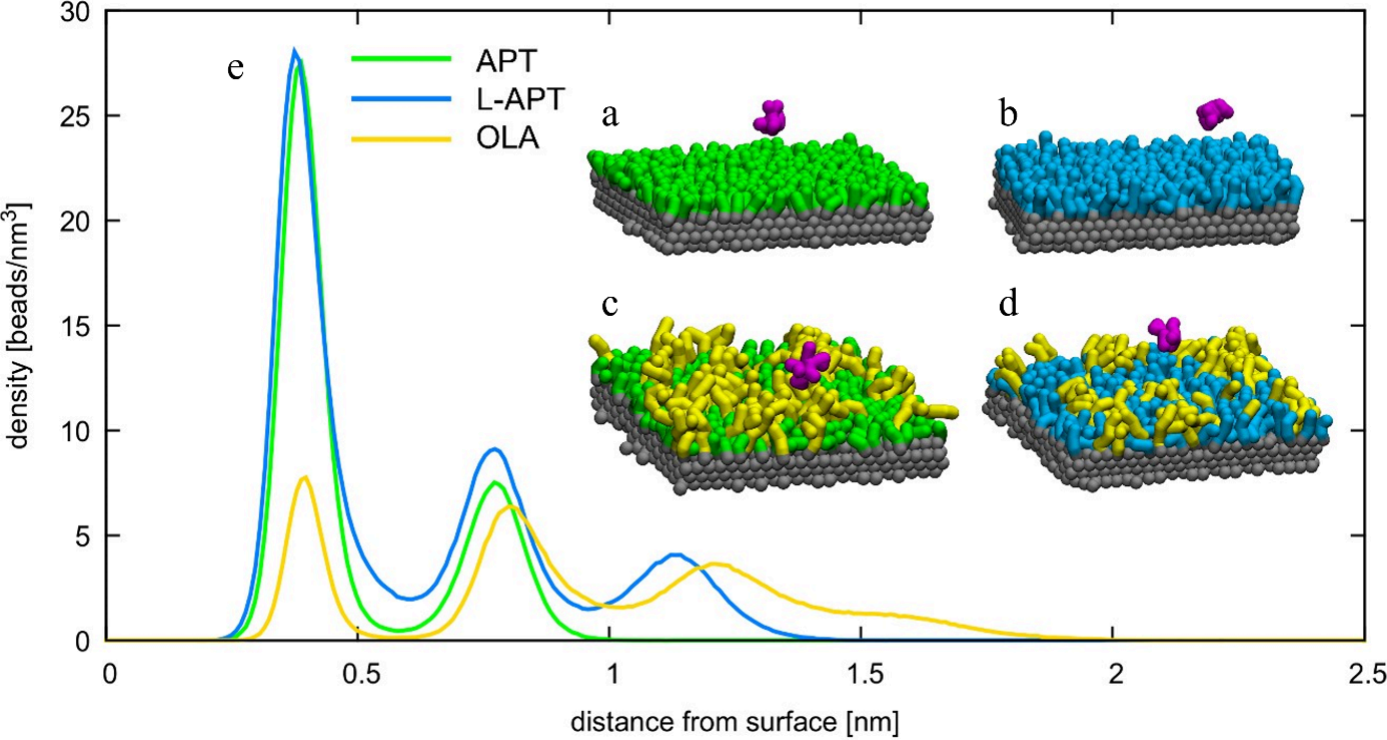
CFZ molecule (purple) plus ZnO surface functionalized with (a) APTMS, (b) APTMS and OLA, (c) L-APTMS, (d) APTMS and OLA. (e) Densities of APTMS, L-APTMS, and OLA as a function of the distance from the surface.

In coarse-grained simulations, four layers of ZnO CG beads organized in a face-centered cubic packing constituted the ZnO matrix (Figure 3) exposing two (111) surfaces to the solvent (water or ethanol) environment. The matrix was maintained unchanged during the simulations because of the high Lennard-Jones interaction strength between ZnO beads. Functional moieties, namely OLA and APTMS, were covalently bound to the surface ZnO beads. The case of L-APTMS will be discussed in the discussion and conclusion sections. Two sets of surface functionalizations were made. The ZnO surface was functionalized with APTMS or OLA+APTMS (see the snapshots as insets in Figure 3). The overall ligand occupation on the functionalized surface layer represented 65% of the total number of beads present on that surface, which is equivalent to a grafting density of 2.65 molecules per nm^2^. The choice of this surface coverage resulted in agreement with that achieved at the all-atom level by OLA molecules only, which corresponded to about 75% of the total NC surface.^29^ The surface area was equal to ∼12 × 12 nm^2^ with a height of 1.5 nm. The functionalized surface and one molecule of CFZ (in magenta; see the snapshots in Figure 3) were placed in a simulation box with a *z* dimension of approximately 18 nm. Water or ethanol were used as solvents for each system. After solvation, NaCl salt was added to water at a physiological concentration of 0.15 molar to mimic the physiological conditions. Figure 3e shows the distribution of APTMS and the OLA plotted against the distance from the solid surface first layer, thus suggesting that the OLA tails extend to a longer distance with respect to APTMS.

### Study of Drug Uptake: Experimental Results

**Figure 4:**
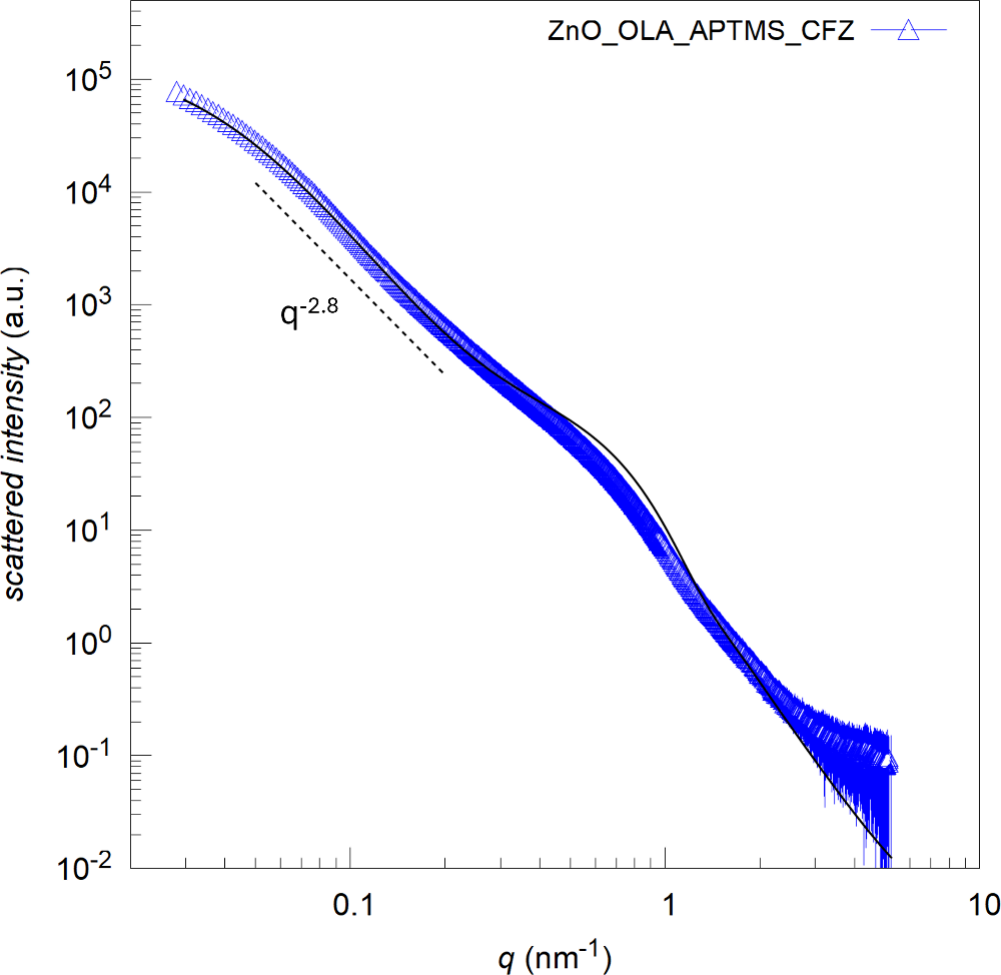
SAXS profiles (symbols) of ZnO nanocrystals with functionalizations upon CFZ loading together with the best fit (black line). The dashed black drives the eye showing the slope of the data at low q-vector values, related to NCs fractal dimension.

We adsorbed CFZ in ethanol by preparing a concentrated solution of CFZ and dispersing the ZnO_OLA_APTMS NCs. However, characterization techniques adopting UV–vis adsorption or HPLC obtained high variability of the results (data not shown). These techniques were indeed not adequate to estimate with confidence the amount of CFZ drug on the pristine ZnO, OLA-functionalized, or dual OLA and APTMS functionalized surfaces. To comprehend the effect, if any, of CFZ uptake on NCs structure, SAXS investigation was performed (Figure 4 and fit parameters in Table 3).

**Table 3:** Fit Parameters of SAXS Data of Functionalized NCs upon CFZ Drug Loading Using a Model of Fractal-Core Shell, by SasView^a^

	radius (nm)	Th_sh_ (nm)	fractal dimension	correlation length (nm)	Bragg spacing (nm)	SLD shell (10^–6^ Å^–2^)
ZnO_OLA_APTMS_CFZ	2.3	0.6	2.85	25	—	9

a Radius (inner core), Th _sh_ = thickness of the shell, SLD _sh_ = SLD of the shell, Bragg spacing = 2π/*q* from peak position (∼1.7 nm^–1^). Scattering length density (SLD) of the core was fixed at 45 × 10^–6^ Å^–2^. The polydispersity of the radius is assimilated to the width of the distribution of ±40%. The uncertainty of the fitting parameters for shell thickness was estimated at ±0.1 nm, for SLD _sh_ at 0.2 × 10^–6^ Å ^–2^, while for the correlation length ±10%.

The main features of the NCs after CFZ uptake are kept (Figure 2d for comparison). Nonetheless, some differences can be spotted, indicating the presence of the drug into the system. First, if compared to the pristine and functionalized NCs in Figure 2d, the CFZ containing sample (Figure 4) shows the highest fractal dimension (related to low q-vector slope), a symptom of a highly correlated system.

Furthermore, the SLD (9 × 10^–6^ Å^–2^) of the shell adsorbed on NCs surfaces increases, suggesting adsorption of the drug into the oleic acid + APTMS layer. This is an expected behavior due to the high hydrophobicity of the drug, even if adsorption could not be ensured in advance. In addition, upon drug adsorption, the Bragg peak showing at *q* ∼ 1.72 nm ^–1^, distinguished in the other NCs SAXS spectra, disappears, indicating that the drug alters the NCs relative interactions. Such findings prove that CFZ is part of the NCs dispersion and suggest that it disposes in the highly hydrophobic portions offered by OLA and APTMS through which nanosized subunits of NCs are connected, breaking the NCs subunits’ preferential orientation in space.

### Free Energy Profiles for CFZ Adsorption on the Functionalized ZnO Surface

To support the interpretation of the experimental results on drug uptake, we used molecular simulations to quantify the free energy of adsorption of CFZ on surfaces functionalized by APTMS only or APTMS+OLA moieties.

**Figure 5:**
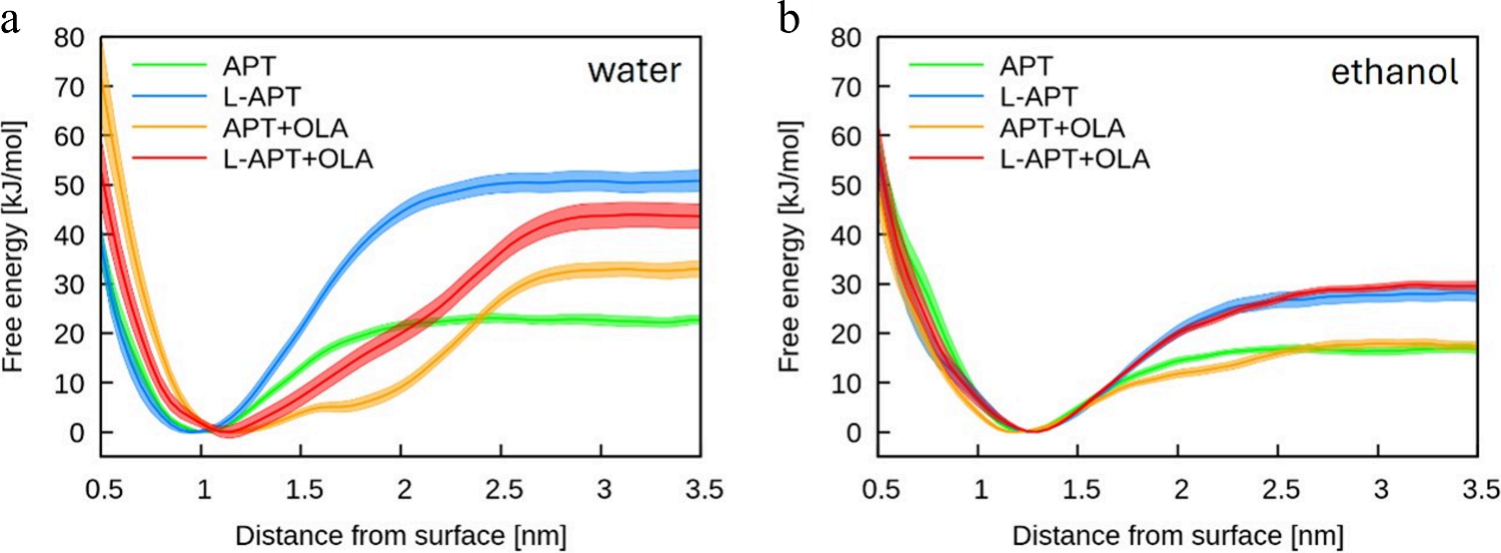
Free energy of adsorption of one CFZ on the ZnO surface (a) in water and (b) in ethanol.

The potential of mean force (PMF) associated with CFZ adsorption was calculated by means of Metadynamics, as described in detail in the Materials and Methods section. Figure 5 shows the PMF profiles of CFZ on the functionalized ZnO surfaces plotted against the distance from the center of mass of the ZnO matrix to the center of mass of CFZ. Error estimates are represented as shaded areas. OLA is important to favor the adsorption of CFZ in the presence of APTMS, increasing the adsorption energy from 20 kJ/mol (APTMS only) to 32 kJ/mol (APTMS+OLA). Moreover, the addition of OLA to the surface results in a shifting of the equilibrium adsorption distance to larger values (from about 0.9 to about 1.1 nm), while also leading to the enlargement of the range of attractive interactions between CFZ and the functionalized surface. The adsorption of CFZ on the NP surface could be responsible for the deactivation of the drug as we have previously shown.^33^ Briefly, the epoxide ring can undergo an opening reaction induced by the presence of water and simultaneous chemisorption on exposed Zn atoms, whose reactivity is especially enhanced on the polar surfaces of the ZnO NCs. This reactive scenario characterizes a regime dominated by a high density of exposed sites on the NCs surfaces, which determines the pinning of the drug molecule via its several nucleophilic ketonic oxygen atoms of the CFZ backbone. Chemisorption can be followed by a protonic transfer from water to the O_epox_ or C_ket_ atoms of CFZ, irreversibly altering the drug’s native structure. RMD singled out an alternative mechanism in which the indirect interaction of CFZ with polar surfaces mediated by a layer of adsorbed water could induce analogous proton transfer reactions. In a previous investigation, we have demonstrated that OLA chains can not be sufficient in passivating ZnO surfaces,^25^ as NCs of about 4 nm in dimension functionalized by OLA chains exhibited uncovered surface regions with polar character on which CFZ could interact via solvent displacing.

In ethanol, on the other hand, the effect of OLA on the adsorption of CFZ is almost negligible. It should be noted that the adsorption energy of CFZ on the APTMS-functionalized ZnO surface in ethanol is about 5 *k*_B_*T*, suggesting a more dynamic interaction of CFZ with the ligand layer than in water.

This is a clear indication, driven by simulation, that the experimental drug adsorption on the ZnO_OLA_APTMS surface has to be carried out in ethanolic solution to prevent drug deactivation.

### Ub-G76V-GFP MM Cells as a Reliable Model To Monitor Proteasome Inhibition

Finally, we conducted in vitro biological assays on MM cells to assess the presence and efficacy of CFZ-loaded NCs. To accurately assess the efficacy of proteasome inhibition, we established two MM cell lines (AMO-1 and KMS-28BM) engineered to express the ubiquitin-fusion degradation substrate Ub-G76V-GFP, a modified version of ubiquitin (G76V) unable to be recognized by deubiquitinases fused to eGFP.^38^ Upon inhibition of the proteasomal proteolysis pathway, this short-lived fusion protein is stabilized, leading to detectable GFP fluorescence.

Initially, AMO-1 and KMS-28BM cells were transduced with lentiviral particles carrying Ub-G76V-GFP, resulting in transduction rates of 30.0% and 39.4%, respectively, as determined by postpuromycin selection at 72 h postinfection (data not shown). Both cell lines fully recovered within 10 days, with over 95% of cells exhibiting low levels of GFP fluorescence, as confirmed by flow cytometry (data not shown). Subsequently, we subjected Ub-G76V-GFP AMO-1 and KMS-28BM cells to increasing concentrations of free drug CFZ (from 0 to 5 nM) and monitored cell viability, GFP positivity, and mean fluorescence at 6, 12, and 24 h post-treatment. Remarkably, GFP mean fluorescence exhibited a progressive increase over time in both cell lines treated with 2.5 and 5 nM CFZ (see Figure S.7 in the Supporting Information). Control experiments conducted using the original AMO-1 and KMS-28BM cell lines demonstrated no significant alterations of mean fluorescence intensity (Figures S.8A,B in the Supporting Information). Consistent with expectations, CFZ treatment did not significantly impact the viability of wild-type (W.T.) or Ub-G76V-GFP AMO-1 and KMS-28BM cells up to 12 h, while cytotoxicity became evident at 24 h with 5 nM CFZ treatment, indicative of functional inhibition of proteasome activity (Figures S7C,D and S.8C,D of the Supporting Information). Overall, these findings confirm the utility of the Ub-G76V-GFP system as a robust method for quantifying proteasome inhibition in MM cell lines and specifically assessing the efficacy of CFZ-loaded NCs.

### Evaluation of Drug Loading on Nanocrystal Surface through the Measurement of Proteasome Inhibition and Cytotoxicity in Multiple Myeloma Cells

To assess the successful loading of CFZ onto the surface of NCs, we directly administered CFZ-loaded NCs to unmodified MM cells.

**Figure 6:**
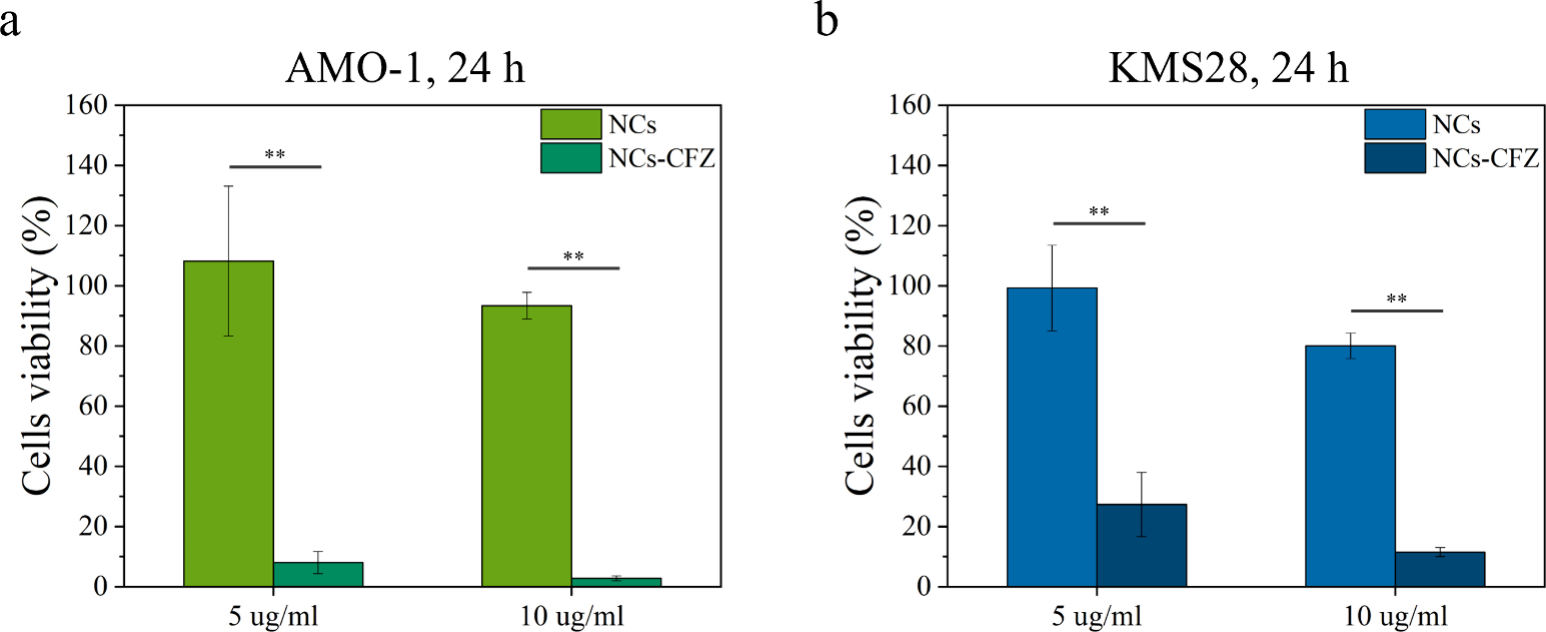
Comparison of the cytotoxic effect of the NCs administered at 5 and 10 μg/mL with and without the drug loaded on their surface on (a) AMO-1 and (b) KMS-28BM cell lines.

The results, presented in Figure 6, indicate that CFZ-loaded NCs induce significant cytotoxic effects in both tested cell lines, particularly in AMO-1 cells (Figure 6a), suggesting efficient drug delivery by the NCs. Comparisons with free CFZ administration data (Figures S.7 and S.8 in the Supporting Information) indicate that the effective concentration of drug released from the NCs exceeds 5 nM. Importantly, viability tests confirm that ZnO_OLA_APTMS NCs alone, without CFZ, are nontoxic at the concentrations tested, suggesting that cell death is due to the drug delivery and not the NCs themselves. Given the strong cytotoxicity observed with drug-loaded NCs at low concentrations, we explored the potential to reduce CFZ loading on the NC surface by performing multiple washes in cell culture medium (Figure S.9 in the Supporting Information). These washes were designed to be compatible with cell viability, as the cell culture medium is water-based, and previous simulations suggest that CFZ has a higher affinity for the functionalized ZnO surface in aqueous environments than in ethanol. The multiple washing steps gradually removed CFZ from the NC surface. This process enables a fine control over NC cytotoxicity while also confirming that CFZ interacts strongly with the functionalized OLA and APTMS surface: more than four washes are required to fully remove CFZ from the NCs, corroborating both simulation and experimental data. Comparisons between these results and the ones derived from free CFZ administration (Figure S.7) suggest that, after four washes, the residual CFZ concentration on the NCs is approximately 2.5 nM or lower (Figure S.8). Internalization tests were also performed via flow cytometry and fluorescence microscopy to confirm the successful uptake of NCs by cancer cells, with the results of these analyses reported in Figures S.10 and S.11 of the Supporting Information.

**Figure 7:**
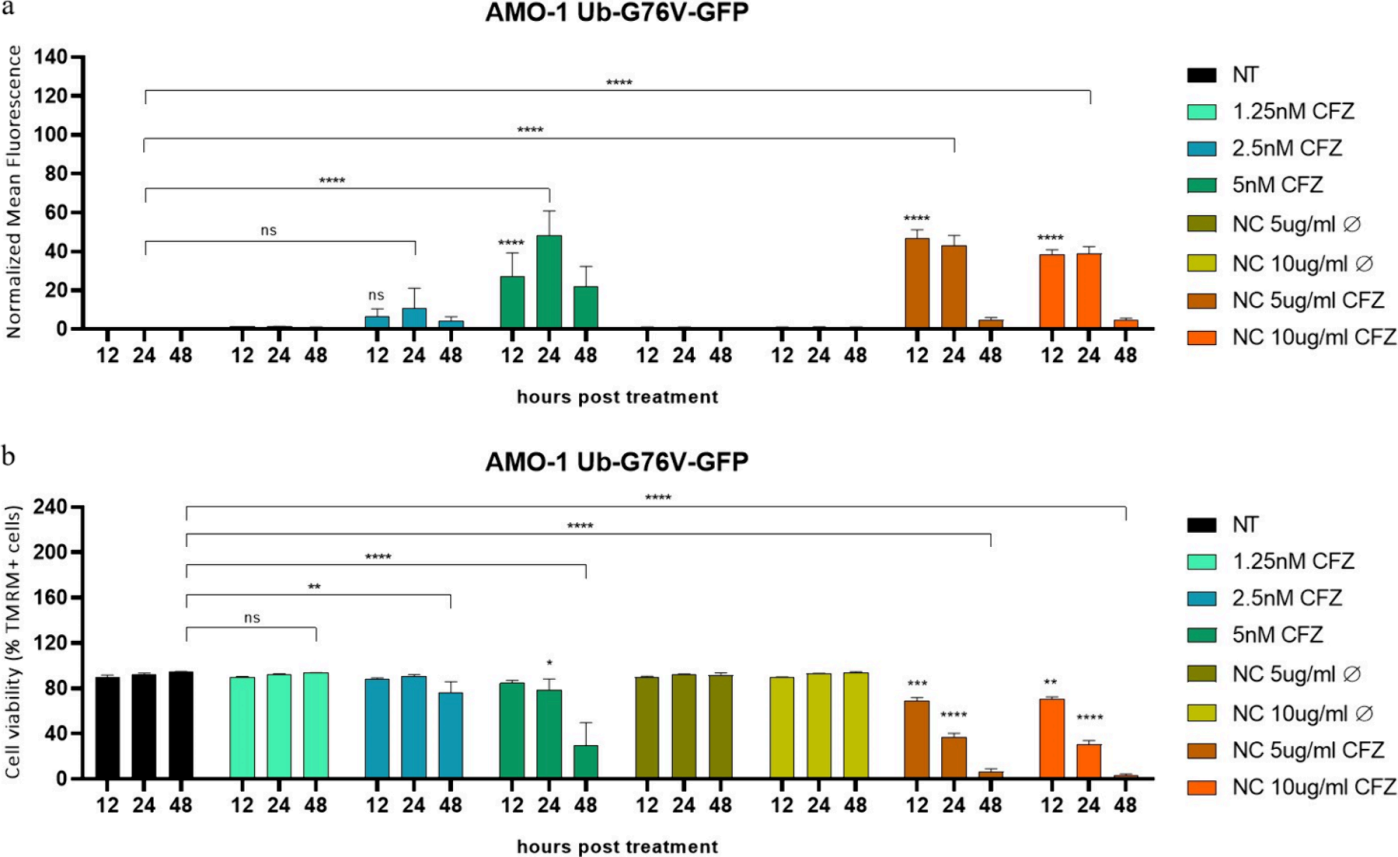
(a) GFP+ mean fluorescence signal of AMO-1 Ub-G76V-GFP with increasing concentration of CFZ (0–1.25–2.5–5 nM) and two different concentrations of ZnO-OLA_APTMS NCs (5 and 10 μg/mL) with and without CFZ (washed three times) at 12, 24, and 48 h post-treatment. Every value was normalized to the respective untreated sample at the indicated time point. (b) Cell viability of AMO-1 Ub-G76V-GFP with increasing concentration of CFZ (0–1.25–2.5–5 nM) and two different concentrations of ZnO-OLA_APTMS NCs (5 and 10 μg/mL) with and without CFZ (washed three times) at 12, 24, and 48 h post-treatment.

To validate the bioactivity of CFZ-loaded NCs, a proof-of-concept experiment was conducted using the engineered Ub-G76V-GFP AMO-1 cell line, which allows measurement of proteasome inhibition through GFP fluorescence. CFZ-loaded NCs, washed three times to reduce cytotoxicity while retaining efficacy, were administered to these cells at 5 and 10 μg/mL. Controls included untreated NCs and free CFZ at incremental concentrations (0, 1.25 2.5, and 5 nM). Cell viability, GFP positivity, and mean fluorescence were monitored at 12, 24, and 48 h post-treatment (Figure 7). The results show a moderate increase in GFP fluorescence in cells treated with 2.5 nM free CFZ, with a stronger response at 5 nM and in all CFZ-loaded NC treatments. Notably, CFZ-loaded NCs led to an enhanced GFP signal as early as 12 h, surpassing the effect of the free drug (Figure 7a). Cell viability data reveal that cytotoxic effects from free CFZ were only significant at 48 h with 2.5 nM and at both 24 and 48 h with 5 nM. In contrast, CFZ-loaded NCs exhibited a marked reduction in cell viability as early as 12 h, particularly evident at 48 h, even at minimal dosages (Figure 7b). A control experiment to assess the preferential therapeutic efficacy toward cancer cells was performed by administering the three times washed, CFZ-loaded NCs (both at 5 and 10 μg/mL) to healthy peripheral mononuclear blood cells (PMBC). The results are reported in Figure S.12 of the Supporting Information.

Taken together, these findings demonstrate that NCs

- successfully adsorb and maintain the pharmaceutical activity of CFZ;
- deliver to cancer cells a biologically effective dose of drug, enabling proteasome inhibition comparable to free CFZ;
- retain an active CFZ concentration on the NC surface after three washes, estimated to be above 5 nM, based on GFP and cell viability analyses;
- do not impact on healthy cells, like peripheral mononuclear blood cells (PBMC).

Collectively, these results validate the therapeutic potential of ZnO_OLA_APTMS NCs as efficient drug carriers for CFZ, demonstrating their capability to maintain drug activity and selectively deliver CFZ to target MM cells. Future experiments should additionally support the targeting capability of these NCs, by adopting specific biomolecule functionalization, selective toward the cell of interest.

### Discussion: A Multiscale, Multifaceted Tiered Approach to Drug Delivery Systems’ Design

DDSs such as the one we have considered here are required to satisfy at one time several compelling requests and can be properly characterized only adopting a wide range of different techniques, addressing not only the characterization of the final construct but also that of the intermediates along the synthesis pathway. Such multistep and multiscale characterization is informative and fundamental for a knowledge-based material design.

To this aim, initial experimental characterizations were performed, allowing us to verify the successful synthesis and the subsequent functionalization of spherical, single-crystalline, iron-doped ZnO NCs, while confirming the pivotal role played by the addiction of OLA and APTMS on both the nanoconstruct stability in solution and mainly in the drug adsorption on the nanocrystals surface.

To further highlight the importance of a multiscale characterization, the role played by molecular simulations within such a synergistic effort deserves to be discussed.

In the development of this multifunctional metal-oxide-based nanoconstruct, molecular simulations have been exploited to provide a mechanistic, atomic, or molecular scale interpretation of the complex mutual interactions taking place within the nanoconstruct. For example, reactive simulations have warned against possible inactivation of the drug on the pristine oxide surface of the NCs.^37^ This warning calls for the experimental quantification of the NCs coverage by functionalizing ligands or the use of specific solvents for drug uptake. Another example of a fruitful interaction between simulations and experiments is provided by the difficulties encountered at the experimental level on the quantification of the degree of adsorption of the drug on the functionalized surface of the NCs. Such a difficulty is explained at microscopic level by the relatively low free energies of adsorption calculated by means of the nanoconstruct’s CG model.

Yet this interplay is, in perspective, not enough. The main advantage constituted by a fruitful feedback loop between experiments and simulations consists in the ability to make informed decisions concerning the design of the nanomaterial. Within an envisaged tiered approach to the design of drug delivery vectors, after the first tier of design → experimental characterization → computational interpretation → in vitro performance assessment, simulations should be exploited to make useful predictions, guiding the refinement of the initially proposed delivery agent and the reinitialization of the loop (see Scheme 1).

In this framework, we propose a simple example of how the second tier may be initialized by computer simulations. The low, undetectable amount of CFZ adsorbed on the nanoconstruct suggests that an increase of the NC’s surface hydrophobicity may favor a better loading. We thus hypothesized the use of an amine-terminated ligand with a longer carbon chain, L-APTMS. We used molecular simulations to characterize the structure of the NCs surface functionalized by L-APTMS and L-APTMS+OLA, and we calculated the free energy of adsorption of CFZ on these two, tier-2 interfaces. As a result, we predicted little to no effect of L-APTMS on the location of the drug on the functionalized surface, but a significant increase of the free energy of adsorption of CFZ on the NC in ethanol (see Figure 5b), which is the medium in which loading is experimentally achieved. In ethanol, OLA has an almost negligible effect on the adsorption of CFZ, which is instead favored by the presence of L-APTMS. The suggestion to use L-APTMS as an alternative to APTMS may then be transferred to the laboratory, where the tier-2 material could undergo characterization and testing.

The results presented in this paper can be considered as a first step toward the standardization of such an envisaged tiered approach.

## Conclusion

This work presents an integrated experimental and computational approach for the development of functionalized zinc oxide nanocrystals (ZnO NCs) as a drug delivery system for the hydrophobic drug carfilzomib (CFZ). By combining wet-chemical synthesis with advanced surface functionalization techniques, we successfully prepared iron-doped ZnO NCs with oleic acid and aminopropyl groups, achieving improved colloidal stability and drug adsorption properties. Comprehensive characterization, including DLS, SAXS, and HR-TEM, revealed the structural and colloidal properties of the NCs, demonstrating the critical role of functionalization in enhancing NC dispersion and drug-loading efficiency. Molecular simulations complemented these experiments by elucidating the adsorption mechanisms and thermodynamics of interactions of CFZ with the functionalized NC surfaces. This synergistic approach enabled us to identify optimal conditions for CFZ loading, mitigating potential drug deactivation, and providing valuable insights into the dynamic interplay between drug molecules and functionalized NC surfaces. Biological assays further validated the efficacy of CFZ-loaded NCs, demonstrating their potential to enhance proteasome inhibition in multiple myeloma cell lines.

Future studies could explore additional functionalizing ligands to fine-tune the surface chemistry of the NCs for CFZ and other hydrophobic drugs or complex therapeutic agents. Looking forward, this methodology lays the foundation for more targeted and efficient nanocarrier designs.

## Supporting Information Available

Molecular dynamics simulations at the coarse-grained level and the related models; additional data and characterizations of the ZnO NCs; characterization of the NCs by small angle X-ray scattering; in vitro model to assess proteasome inhibition sensitivity of MM cells (PDF)
