# Supplementary material for: Diving on the Surface of a Functional Metal Oxide through a Multiscale Exploration of Drug–Nanocrystal Interactions
Source: ACS Appl Mater Interfaces. 2025 Feb 10;17(7):10432–45. doi: 10.1021/acsami.4c19916 (PMC11843534; doi:10.1021/acsami.4c19916)
Supplement: Supplementary file 1 — am4c19916_si_001.pdf [file am4c19916_si_001.pdf]

Supporting Information

Diving on the surface of a functional metal oxide through a multiscale exploration of drug-nanocrystal interactions

Nicolò Maria Percivalle[1¥], Julia Blandine Bassila[2¥], Alice Piccinini[3¥], Michela Cumerlato[4], Mariangela Porro[4], Cheherazade Trouki[5,6], Susanna Monti[7], Giovanni Barcaro[5], Davide Bochicchio[2], Roberto Piva[4], Valeria Rondelli[3], Giulia Rossi[2*], Valentina Cauda[1*]*

[1] Department of Applied Science and Technology, Politecnico di Torino, Corso Duca degli Abruzzi 24, 10129 Turin, Italy

[2] Department of Physics, Università degli Studi di Genova, Via Dodecaneso 33, 16146 Genoa, Italy

[3] Department of Medical Biotechnology and Translational Medicine, Università degli Studi di Milano, L.I.T.A., V.le F.lli Cervi 93, 20054 Segrate, Italy

[4] Department of Molecular Biotechnology and Health Sciences, University of Turin, Piazza Nizza 44, 10126 Turin, Italy

[5] CNR-IPCF, Institute for Chemical and Physical Processes, Via G. Moruzzi 1, 56124 Pisa, Italy

[6] Department of Pharmacy, University of Pisa, Via Bonanno 6, 56126 Pisa, Italy

[7] CNR-ICCOM, Institute of Chemistry of Organometallic Compounds, Via G. Moruzzi 1, 56124 Pisa, Italy

¥ These authors contributed equally to the work.

**Corresponding authors:*

Prof. Valentina Cauda, phone: +390110907389, e-mail: valentina.cauda@polito.it

Prof. Giulia Rossi, phone: +390103536239, e-mail: giulia.rossi@unige.it

Prof. Valeria Rondelli, phone +390250330323, e-mail valeria.rondelli@unimi.it

1. Molecular Dynamics Simulations at the Coarse-grained level and the related models

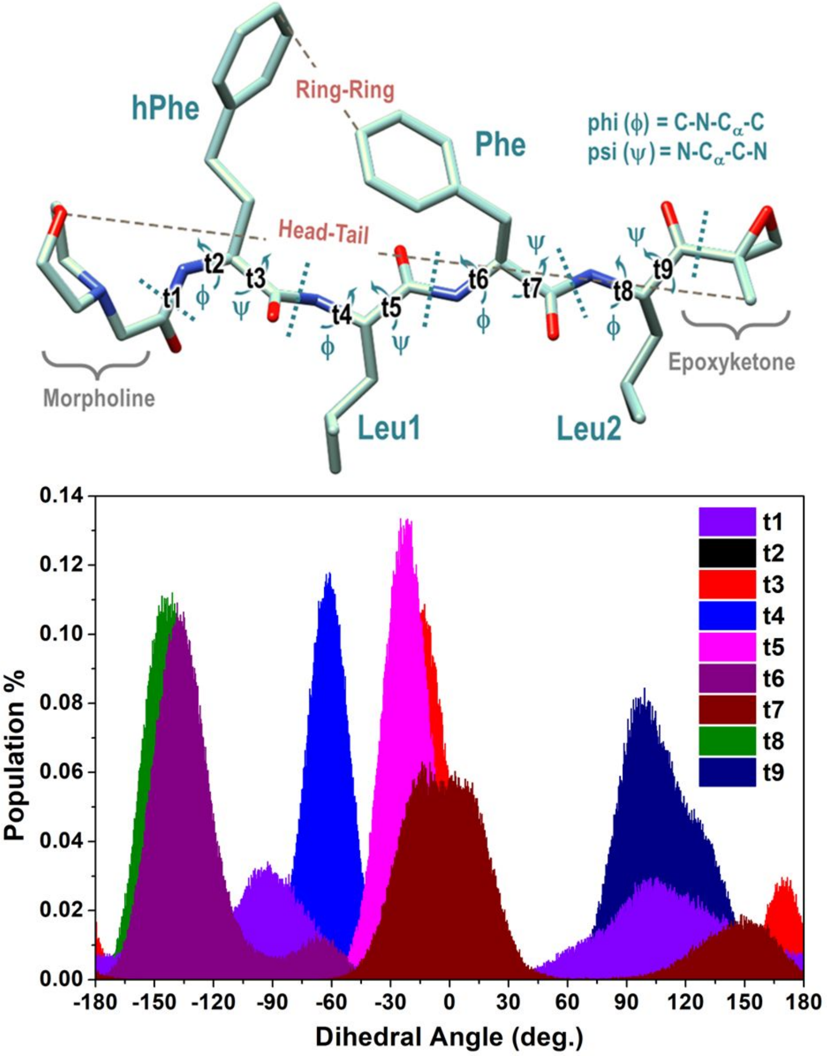

Figure S.1. **Top.** CFZ structure (hydrogens are undisplayed). **Bottom.** Distributions of the dihedral angles of CFZ: t1 = C-NC-C angle, t2-t9 = phi and psi dihedral angles of all the residues (hPhe, Leu1, Phe, Leu2).

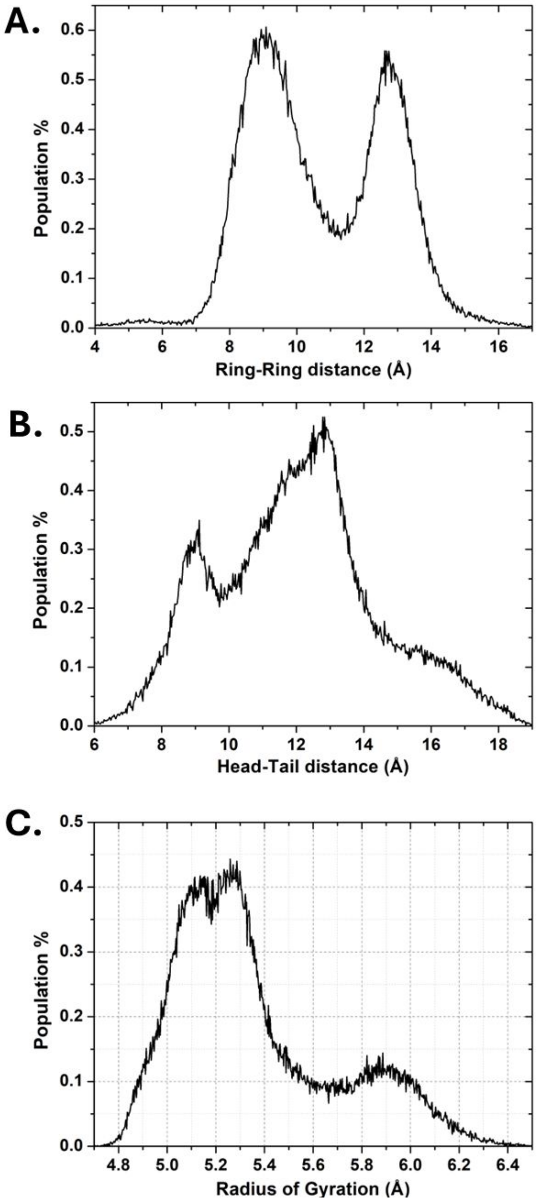

Figure S.2. Distributions of various structural descriptors of CFZ obtained from the All-atom MD sampling in water solution (50 ns). **A.** Ring-ring distance. **B.** Head-tail distance. **C.** Radius of gyration.

The mapping of CFZ and the choice of the Martini beads to represent it are shown in Figure S.1a. P2 beads were used to represent the peptide backbone, which is recommended for Martini 3 proteins. This choice and that of the other bead types was validated by performing thermodynamic integration (T.I.) simulations to calculate the water-octanol partitioning of CFZ. A free energy of transfer $\Delta G_{OW} = 24.0 \pm 2.5$ kJ/mol was obtained, which is in excellent agreement with the logP of 4.6 reported in the Kyprolis (CFZ) Safety Data Sheet (corresponding to $\Delta G_{OW} = 26.3$ kJ/mol). Bonded interactions have been fitted to atomistic data from the all-atom molecular dynamics simulations previously discussed. After a first guess obtained with the SWARM-CG tool[1], the interactions have been manually refined. In Figure S.2, we show the bond and angle distributions of 1 coarse-grained CFZ molecule in water compared with the same distributions obtained from 50 ns of atomistic trajectory.

To represent ZnO, which is a hydrophilic material, a polar (P1) bead was selected, while to ensure the stability of the solid ZnO matrix, the ZnO-ZnO ε parameter of the Lennard Jones potential was set to a high value, 40 kJ/mol. OLA (Figure S.3b) was made of four regular hydrophobic (C1 and C4h) beads to describe its hydrophobic region and a regular charged (Q5n) bead to represent the carboxylate (COO^-) group in the oleate form of oleic acid. It has been reported by Špačková *et al.*[2] that the oleate form of oleic acid is the most prominent form present on ZnO NPs (nanorods) surfaces. The authors used NMR characterization to show that, even at high temperatures, oleate has a strong interaction with ZnO surface through coordination bonds between Zn^{2+} and the carboxylate. They observed only a few weakly bound species interacting through hydrogen bonding as one may expect when the oleic acid form is grafted.

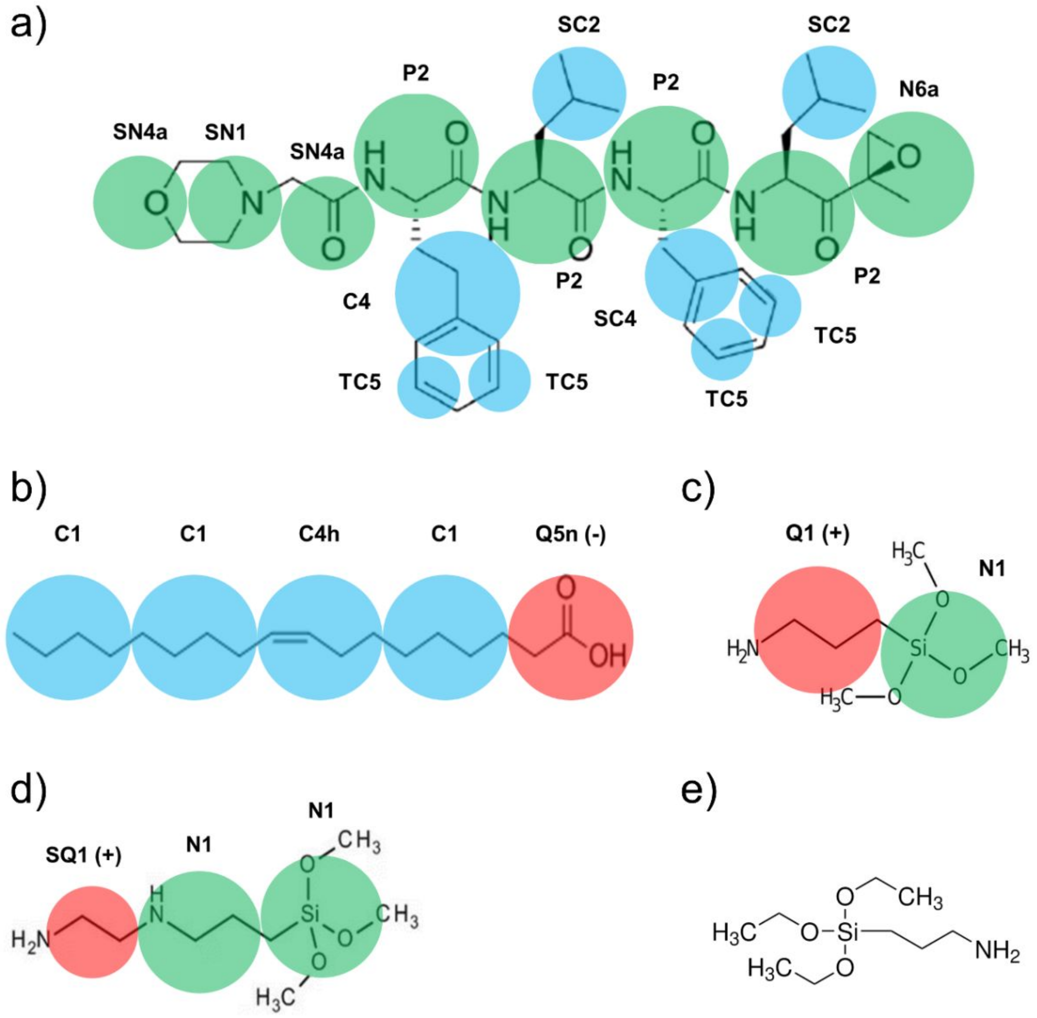

Figure S.3. Atomistic representation and superimposed coarse-grained models of (a) CFZ, (b) OLA, (c) APTMS, (d) L-APTMS, and atomistic representation of (e) APTES. Beads size refers to the number of heavy atoms they are made of; the bead with large size contains four heavy atoms, the medium size contains three heavy atoms, and the small size bead has two heavy atoms. The colors highlight the degree of hydrophobicity; the red beads are charged, the green beads have some degree of polarity, and the blue beads are hydrophobic.

APTMS (Figure S.3c) is represented by two regular beads: the first one is the Q1 bead for the group NH_3^+-CH_2-CH_2-CH_2, while the second one is the N1 bead which represented the group Si-CH_3O-CH_3O-CH_3O. A positively charged Q1 bead was used, since in solution at physiological pH APTMS is expected to be protonated. The chemical structure of L-APTMS (Figure S.3d) is the structure of APTMS plus an additional CH_2-CH_2-NH between the NH_2 amine group and the rest of the atoms, and three beads were selected to map it. The first one is the small SQ1 bead to map the NH_3^+-CH_2-CH_2 group, the second one is the regular N1 bead for the group NH-CH_2-CH_2-CH_2 and the last one is the regular N1 bead to represent the group Si-CH_3O-CH_3O-CH_3O, which is the same used in APTMS.

The choice of a regular N1 bead to model the group Si-CH_3O-CH_3O-CH_3O, composed of seven non-hydrogen atoms in APTMS and L-APTMS, was motivated by the work done by Chandran, A. M. *et al.*[3]. In their work, oxidized surfaces of ZnO NPs were functionalized with APTES (Figure S.3e) in an ethanol medium. The interaction between the APTES molecules and the oxidized ZnO NPs, only mediated by APTES oxygen atoms, lead to the loss of their methyl (CH_3) and methylene (CH_2) groups. This implied that the group Si-CH_3CH_2O-CH_3CH_2O-CH_3CH_2O, which consisted in ten non-hydrogen atoms before the reaction, was left with only four non-hydrogen (Si-O-O-O) atoms after it. As shown in Figures S.3c and S.3d, APTMS and L-APTMS lack three methylene groups in their structure compared with APTES; apart from that, their respective overall chemical structures are identical.

2. Additional data and characterizations of the ZnO NCs

To better explore the use of ZnO_OLA_APTMS NCs as therapeutic nanotools, the stability of such nanocrystals was evaluated in physiologically relevant conditions by performing DLS measurements over time in RPMI and RPMI complemented with 10% FBS (cRPMI), with the latter being the cell culture medium used for all the *in vitro* tests. The results of these measurements are shown in Figure S.4.

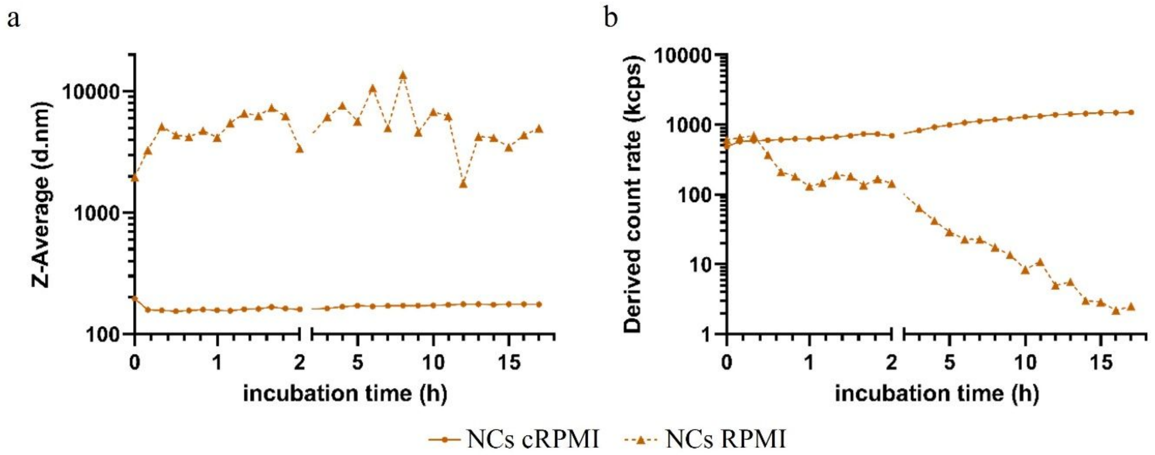

Figure S.4. (a) Z-Average and (b) Derived count rate from repeated DLS measurements over time for NCs in RPMI and RPMI complemented with 10% FBS (cRPMI).

Time-resolved measurements of ZnO_OLA_APTMS NCs in cell culture medium without FBS clearly revealed a phenomenon of particle aggregation as particles reached micrometer-scale dimensions from the first measurement (Figure S.4a). Additionally, the Derived Count Rate data indicated that as the particles aggregated, they precipitated, leading to a progressive decrease in signal

intensity (Figure S.4b). In contrast, the presence of 10% FBS significantly enhanced the stability of the nanoparticles, which maintained stable average size and derived count rate for the entire duration of the measurement. As widely reported in the literature, this can be likely attributed to the adsorption of proteins from the FBS, forming a protein corona which can sterically stabilize the NPs preventing their aggregation.

Crystallographic plane	Diffraction angle
(100)	31.8°
(002)	34.6°
(101)	36.3°
(102)	47.8°
(101)	56.4°

Table S.1. XRD diffraction angles of iron-doped ZnO_OLA_APTMS NCs and the relative crystallographic planes.

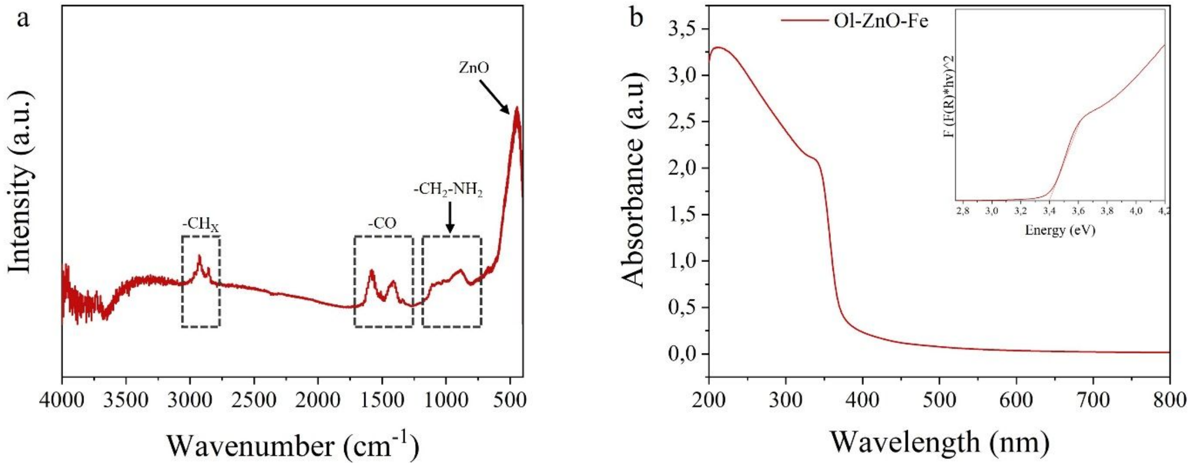

Figure S.5. (a) Fourier transform infrared spectroscopy spectrum of ZnO_OLA_APTMS NCs. (b) UV-visible spectrum of ZnO_OLA_APTMS NCs. The graph in the upper right square represents the Tauc's plot for the evaluation of the optical band gap (Eg).

3. Characterization of the NCs by Small Angle X-ray Scattering

Description of the employed fitting model. SAXS data were fitted with a model of primary core-shell particles, which aggregate into a mass fractal[4].

The Intensity of scattering, in general, can be described by:
$I(Q) \propto P(Q)S(Q) + background$

Where P(Q) is the form factor of a core-shell sphere, described by:

$$P(Q) = \left(\frac{\emptyset}{V_s}\right)\left[3V_c(\rho_c - \rho_s)\left(\frac{\sin(qr_c)\, qr_c \cos(qr_c)}{(qr_c)^3}\right) + 3V_s(\rho_s - \rho_{solv})\left(\frac{\sin(qr_s) - qr_s \cos(qr_s)}{(qr_s)^3}\right)\right]^2$$

While the structure factor is indicated with S(Q) and is represented by:

$$S(Q) = 1 + \frac{\left(D_f\,\Gamma\left(D_f - 1\right)\right)}{\left[1 + \frac{1}{(q\varepsilon)^2}\right]^{(D_f-1)/2}}\left(\frac{\sin\left[\left(D_f - 1\right)\tan^{-1}(q\varepsilon)\right]}{qr^{D_f}}\right)$$

$\emptyset$ is the volume fraction of the particles, V_c is the volume of the core, and V_s is the volume of the whole particle. ρ_c, ρ_s and ρ_{solv} are the scattering length density of the core, shell and solvent. R_c and R_s are the core and whole particles' radii. D_f is the fractal dimension and ε is the correlation length. The model considers the polydispersity of the radius and the thickness employing a gaussian distribution model.

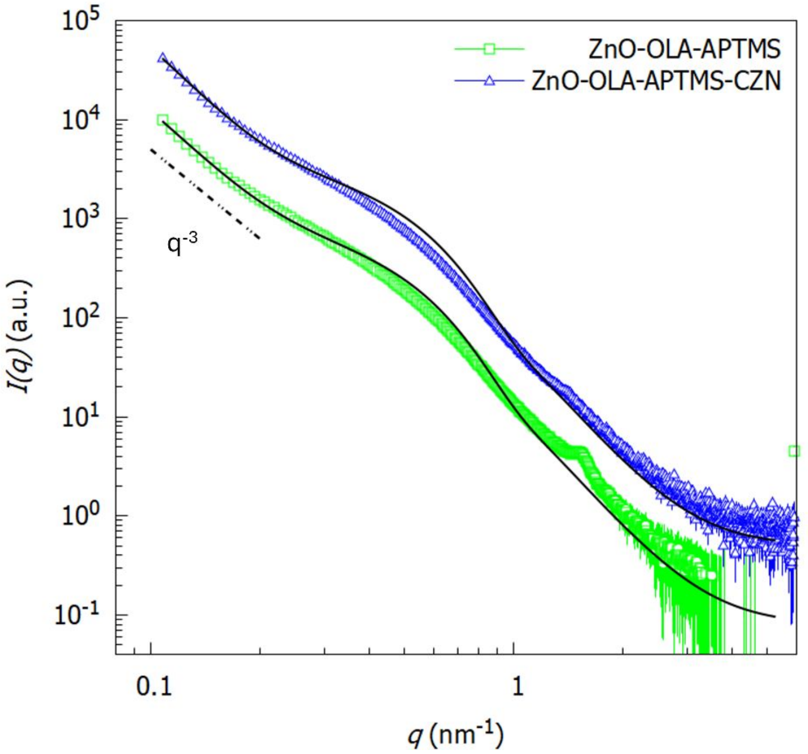

Figure S.6. SAXS data collected at ELETTRA facility: In green ZnO_OLA_APTMS, in blue ZnO_OLA_APTMS_CFZ.

Sample	Radius (nm)	Shell Thickness (nm)	(SLD$_{sh}$ (x10^{-6} Å^{-2})	Fractal dimension	Correlation length	Repeat Distance (nm)
**ZnO_OL_APTMES**	3.00	5.0	8.5	3	25	4.16
**ZnO_OL_APTMES_CFZ**	3.00	6.0	9.0	3.1	22	4.27

Table S.2. Fit parameters of SAXS data collected at ELETTRA, using a model of fractal-core shell, by SasView. Radius (inner core), Th sh=thickness of the shell, SLD sh=SLD of the shell, Fract= Fractal dimension, Peak position=Bragg peak position, Distance= distance calculated with 2π/q from the Bragg peak position. SLD of the core was fixed at 45 x10^{-6} Å^{-2} Polydispersity of the Radius of 0.4.
The uncertainty on the radius is assimilated to the width of the distribution at ± 40%. The uncertainty on the thickness was estimated at □ 0.1 nm. for SLD$_{sh}$ at 0.2 x10^{-6} Å^{-2}, for the fractal dimension 0.02 while for the correlation length ± 10 %.

Chemical compound	SLD (x10^{-6} Å^{-2})
**ZnO - Fe**	45.3
**OLA**	8.5
**APTMES**	12.3
**CFZ**	9.3

Table S.3. SLD values employed for SAXS data fits. Molecular volumes for SLDs calculations are from literature[5,6].

4. In vitro model to assess proteasome inhibition sensitivity of MM cells

Initially, AMO-1 and KMS-28BM cells were transduced with lentiviral particles carrying Ub-G76V-GFP, resulting in transduction rates of 30.0% and 39.4%, respectively, as determined post-puromycin selection at 72 hours post-infection (data not shown). Both cell lines fully recovered within 10 days, with over 95% of cells exhibiting low levels of GFP fluorescence, as confirmed by flow cytometry (data not shown). Subsequently, we subjected Ub-G76V-GFP AMO-1 and KMS-28BM cells to increasing concentrations of free drug CFZ (0 - 1.25nM - 2.5nM and 5nM) and we monitored cell viability, GFP positivity, and mean fluorescence at 6h, 12h, and 24h post-treatment (see Figure S.7). Remarkably, GFP mean fluorescence exhibited a progressive increase over time in both cell lines treated with 2.5 and 5 nM CFZ, as detected by flow-cytometry (Figures S.7A-B) and microscopy (Figure S.7E). Particularly noteworthy was the substantial increase of GFP mean fluorescence intensity relative to untreated samples, observed 24 hours post-treatment with 5 nM CFZ in Ub-G76V-GFP AMO-1 (80-fold increase) and KMS-28BM (20-fold increase) cells. Control experiments conducted using the original AMO-1 and KMS-28BM cell lines demonstrated no significant alterations of mean fluorescence intensity (see Figures S.8A-B). Consistent with expectations, CFZ treatment did not significantly impact the viability of wild-type (W.T.) or Ub-G76V-GFP AMO-1 and KMS-28BM cells up to 12 hours, while cytotoxicity became evident at 24 hours with 5 nM CFZ treatment, indicative of functional inhibition of proteasome activity (Figures S.7C-D and Figures S.8C-D). Overall, these findings confirm the utility of the Ub-G76V-GFP

system as a robust method for quantifying proteasome inhibition in MM cell lines and specifically to assess the efficacy of CFZ-loaded NCs.

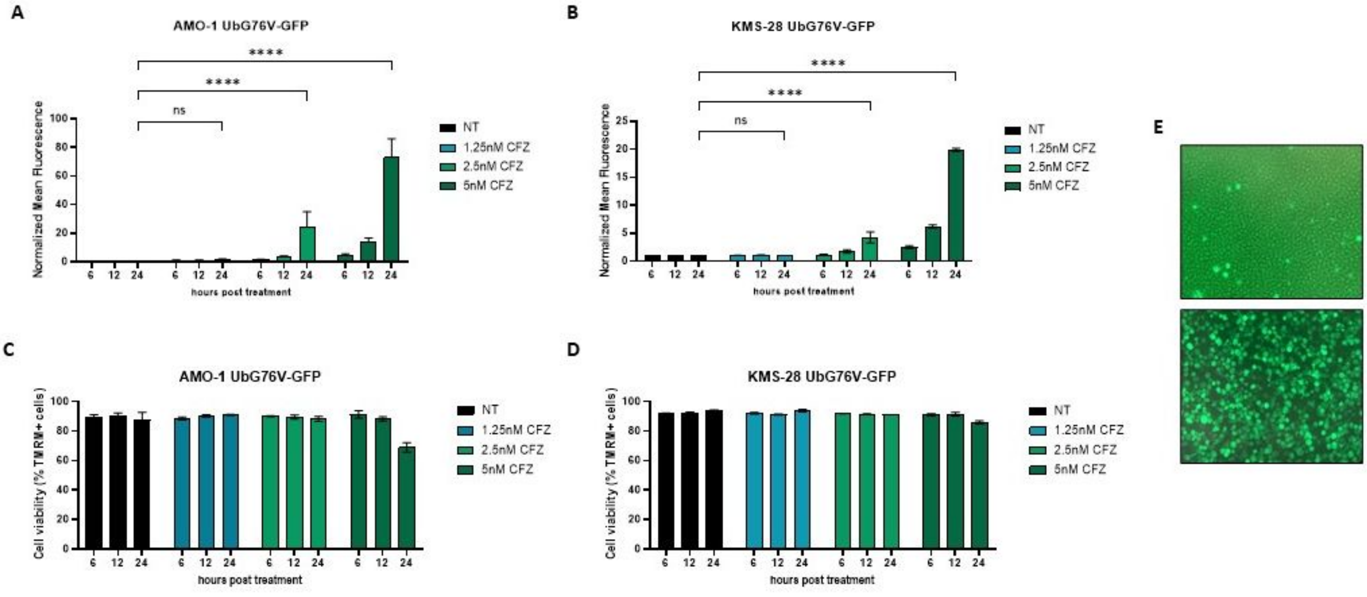

Figure S.7. GFP+ mean fluorescence signal of **A** AMO-1 and **B** KMS-28BM Ub-G76V-GFP with increasing concentration of CFZ (0-1.25-2.5-5 nM) after 6-12 and 24 hours post treatment. Every value was normalized to the respective untreated sample at the indicated time point. Cell viability of **C** AMO-1 and **D** KMS-28BM Ub-G76V-GFP with increasing concentration of CFZ (0-1.25-2.5-5 nM) after 6-12 ad 24 hours post treatment. **E** Representation of AMO-1 Ub-G76V-GFP at 0 (upper) and 5 (lower) nM CFZ, 24 hours post treatment. Data is the means of three independent experiments. Asterisks denote statistical significance (****P<0.0001 or ns>0,05).

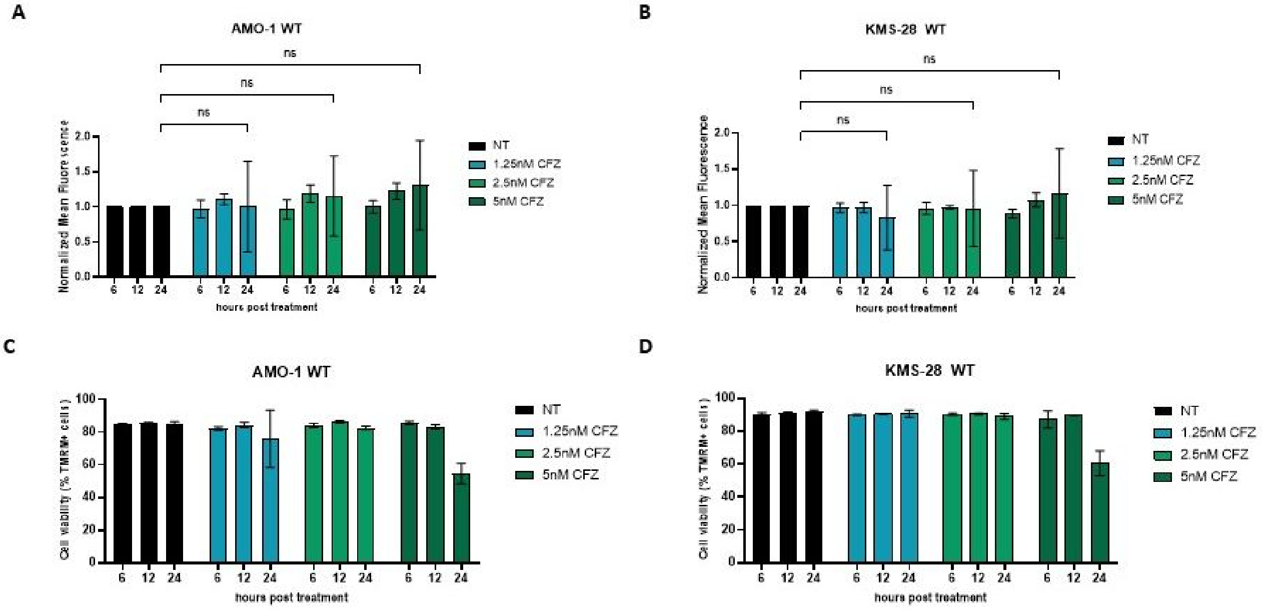

Figure S.8. GFP+ mean fluorescence signal of **A** AMO-1 WT and **B** KMS-28BM WT with increasing concentration of CFZ (0-1.25-2.5-5 nM) after 6-12 and 24 hours post-treatment. Every value was normalized to the respective untreated sample at the indicated time point. Cell viability of **C** AMO-1 WT and **D** KMS-28BM WT with increasing concentration of CFZ (0-1.25-2.5-5 nM) after 6-12 ad 24 hours post-treatment. Data is the means of three independent experiments. Asterisks denote statistical significance (ns>0,05).

Given the extremely high cytotoxicity of the drug-loaded nanocrystals already at really low concentrations, it was interesting to investigate the possibility to reduce the amount of Carfilzomib loaded on the NCs surface by washing them multiple times in cell culture medium and evaluate the cytotoxic potential of the drug present in the supernatants resulting from each washing step (Figure S.9). This washing step was chosen as on the one hand it provides to maintain the NCs in cell growth media, useful to not impart cell viability, on the other hand cell culture media is a water-based solution and from the simulations above it is clear that the drug has a larger affinity for the ZnO functionalized surface in water than in ethanol.

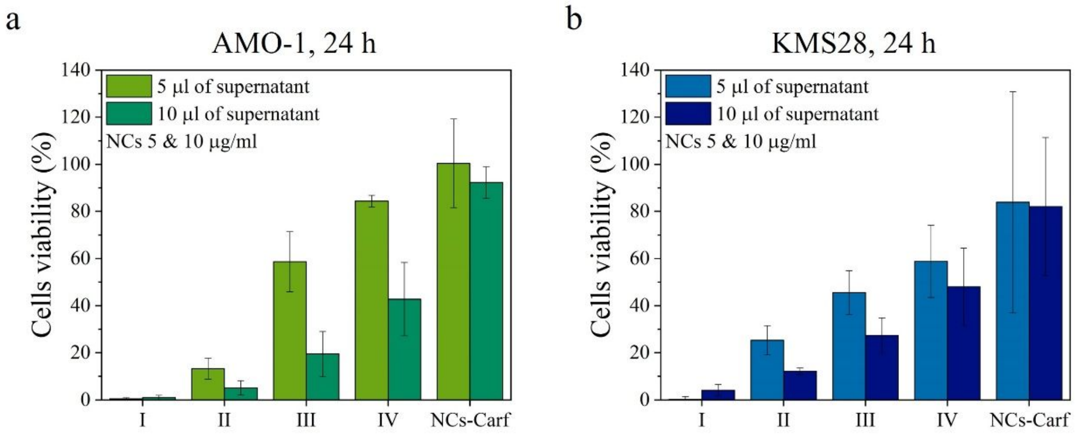

Figure S.9. Cytotoxicity profiles of the supernatants derived from ZnO_OLA_APTMS_CFZ and from the NCs themselves (after 4 washing steps) on (a) AMO-1 and (b) KMS28 cells lines after 24h.

The results of these tests led to different interpretations and possible insights. The first one is that the supernatant derived directly from the drug uptake process (indicated in the graphs with the roman numeral I) is extremely cytotoxic. This is, however, quite obvious given the amount of drug used during the uptake procedure (1mg/ml). A more interesting aspect that can be inferred from the graphs analysis is that the washing steps performed after the drug uptake led to the removal of Carfilzomib from the NCs surface in a step-by-step process. This process on one side allows to better control the cytotoxicity of the NCs, and on the other side, it suggests that the CFZ is interacting firmly with the functionalized OLA and APTMS surface as more than four washing steps are necessary to completely remove the drug from the nanocrystals, confirming the simulation and experimental findings. These considerations are supported by the results in Figure S.8, since both cell lines viability is enhanced at increasing numbers of washing steps, up to the fourth step.
Finally, the last columns at the right side in the graph report the cell viability when administering the ZnO_OLA_APTMS_CFZ NCs after four washing steps. The good cell viability implies that little to no drug was left on the NCs surface. Considering the data of free CFZ administration to both cell lines, as reported in Figure S.6, here again we can roughly estimate that the amount of CFZ left on the NCs surface after four washing steps is below or equal to 2.5 nM. The high value of the standard deviation in the case of KMS-28BM (Figure S.9b) suggests however to be highly cautious with this statement in the case of this cell line.

To assess the successful internalization of NCs inside MM cells, flow cytometry and fluorescence microscopy analyses were performed. For the flow cytometry tests, AMO-1 and KMS28 cells were seeded at $1x10^5$ cells/ml in 24 well-plates and treated with a concentration of 5 and 10 µg/ml of medium of ATTO647-labeled NCs (1 µl of ATTO647 for 500 µg of NCs). After 24h, cells were washed twice with PBS through centrifugation, resuspended in 500 µl of PBS and analyzed.

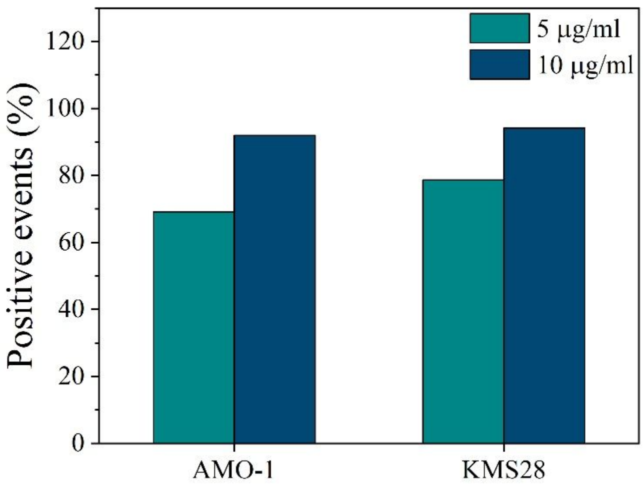

Figure S.10. NCs internalized or attached to AMO-1 and KMS28 cell membranes 24h after treatment.

The results (Figure S.10) consider as positive events both the NCs which are inside cells and the ones bound to the external cellular membranes. The data show effective NCs internalization values for either cell line already after 24h for both NCs concentrations, with an increase of cellular uptake in the case of 10 μg/ml treatment.

To further corroborate these results, fluorescence microscopy analyses were performed. In this case, the same seeding and NCs labeling procedure was carried out, and after 24h, prior to microscopy observation, cells were labeled as follows. First, they were centrifuged and the pellet was resuspended in 250 μl of PBS. To label cell membrane, 0.6 μl of WGA conjugated with Alexa Fluor 488 (WGA488, λex = 495 nm, Thermo Fisher) was added and let incubate for 10 min. Cells were then centrifuged again and resuspended in 250 μl of PBS, and 0.08 μl of Hoechst (Thermo Fisher Scientific) was added to label cell nuclei before other 5 minutes of incubation. Then, after two washing steps with PBS, cells were resuspended in 200 μl of PBS and a 50 μl droplet of this cell solution was spotted in a 8-well chamber slide (Thermo Scientific Nunc Lab-Tek II CC2 Chamber Slide System) for confocal fluorescence microscopy analysis.

\

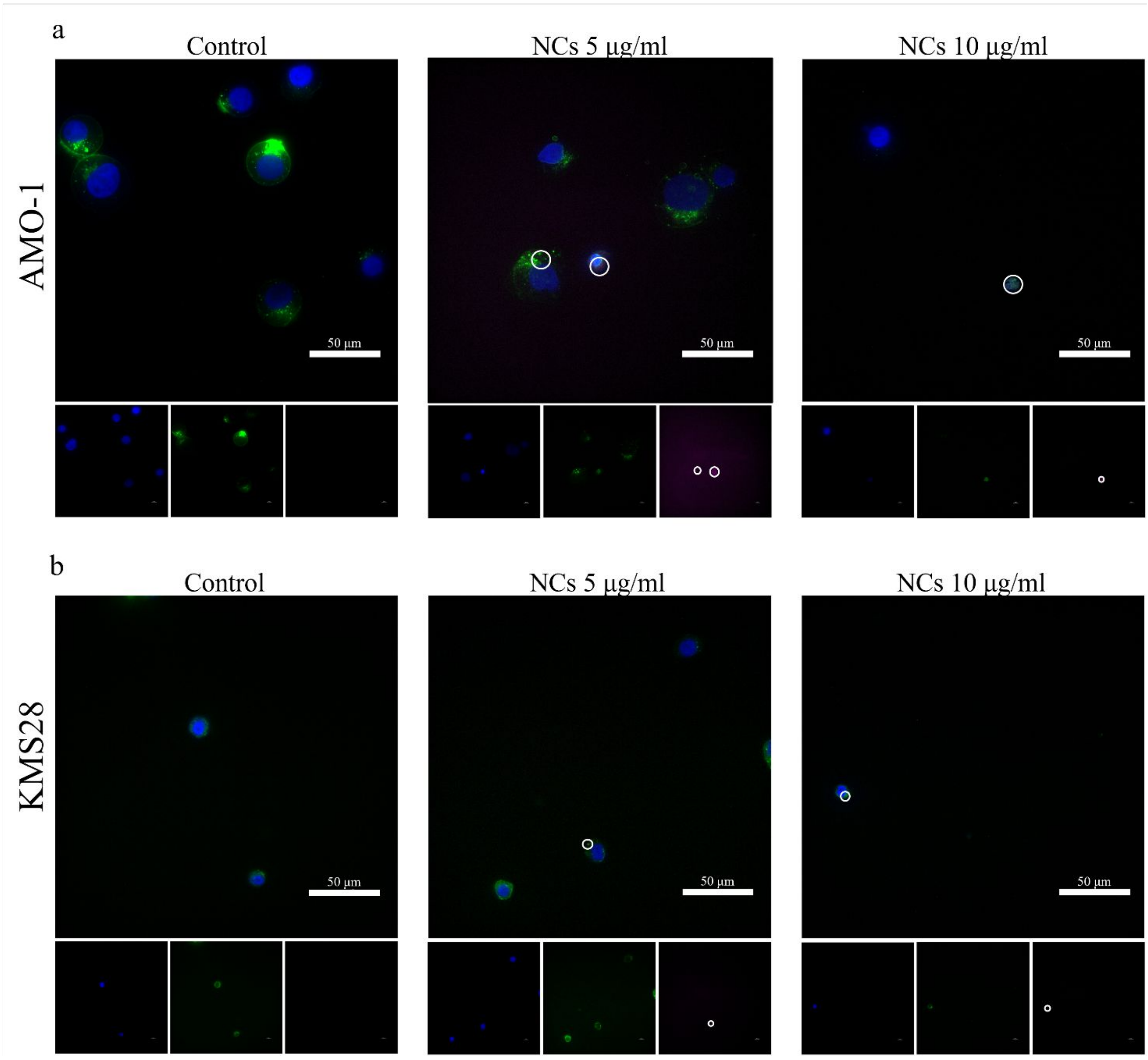

Figure S.11. Fluorescence microscopy images of (a) AMO-1 and (b) KMS28 cells.

Fluorescence microscopy images resulting from these tests (Figure S.11) confirm the internalization of both tested concentrations of NCs (purple elements) inside AMO-1 and KMS28 cells, whose nuclei (blue elements) and membranes (green elements) were labeled.

Finally, a control experiment to assess the preferential therapeutic efficacy towards cancer cells was performed by administering the three times washed, CFZ-loaded NCs (both at 5 and 10 μg/ml) to Peripheral Mononuclear Blood Cells (PMBC) derived from healthy patients. To this aim, cells were seeded at $1x10^6$ cells/ml (1 ml) in 24 well-plates and treated with a concentration of 5 and 10 μg/ml of medium of CFZ-loaded NCs. After 24 and 48 hours, 2.5 μl of a 1 mg/ml solution of Propidium Iodide (PI, ThermoFisher) was added to the cell culture medium, and the samples were analyzed through flow cytometry after 5 minutes of incubation.

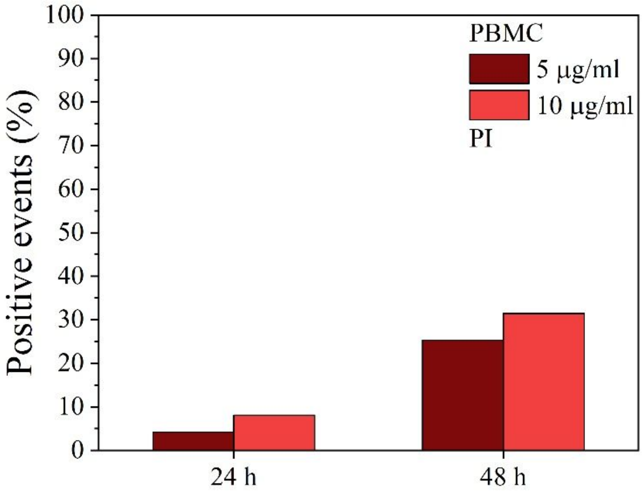

Figure S.12. Membrane integrity assessment of healthy PBMC through PI labeling.

Figure S.12 shows the results in terms of % of positive cells to PI, thus % of dead cells. In particular, it is clear that CFZ-loaded NCs have a limited cytotoxic effect on PBMC cells compared to MM cell lines (Figure 7 in the main text). While a disruption in PBMC membrane integrity, which is directly related to cell death, is noticeable, as well as being time and dose dependent, data at 24 and 48 hours indicate that such cytotoxic effect is rather modest, confirming the preferential therapeutic efficacy towards cancer cells of CFZ-loaded NCs.

References

(1) Empereur-Mot, C.; Pesce, L.; Doni, G.; Bochicchio, D.; Capelli, R.; Perego, C.; Pavan, G. M. *Swarm-CG*: Automatic Parametrization of Bonded Terms in MARTINI-Based Coarse-Grained Models of Simple to Complex Molecules *via* Fuzzy Self-Tuning Particle Swarm Optimization. *ACS Omega* **2020**, *5* (50), 32823–32843. https://doi.org/10.1021/acsomega.0c05469.

(2) Špačková, J.; Fabra, C.; Mittelette, S.; Gaillard, E.; Chen, C.-H.; Cazals, G.; Lebrun, A.; Sene, S.; Berthomieu, D.; Chen, K.; Gan, Z.; Gervais, C.; Métro, T.-X.; Laurencin, D. Unveiling the Structure and Reactivity of Fatty-Acid Based (Nano)Materials Thanks to Efficient and Scalable ^{17}O and ^{18}O-Isotopic Labeling Schemes. *J. Am. Chem. Soc.* **2020**, *142* (50), 21068–21081. https://doi.org/10.1021/jacs.0c09383.

(3) Chandran, A. M.; Varun, S.; Karumuthil, S. C.; Varghese, S.; Mural, P. K. S. Zinc Oxide Nanoparticles Coated with (3-Aminopropyl)Triethoxysilane as Additives for Boosting the Dielectric, Ferroelectric, and Piezoelectric Properties of Poly(Vinylidene Fluoride) Films for Energy Harvesting. *ACS Appl. Nano Mater.* **2021**, *4* (2), 1798–1809. https://doi.org/10.1021/acsanm.0c03214.

(4) Teixeira, J. Small-Angle Scattering by Fractal Systems. *J. Appl. Crystallogr.* **1988**, *21* (6), 781–785. https://doi.org/10.1107/S0021889888000263.

(5) Schindler, T.; Schmiele, M.; Schmutzler, T.; Kassar, T.; Segets, D.; Peukert, W.; Radulescu, A.; Kriele, A.; Gilles, R.; Unruh, T. A Combined SAXS/SANS Study for the in Situ Characterization of Ligand Shells on Small Nanoparticles: The Case of ZnO. *Langmuir* **2015**, *31* (37), 10130–10136. https://doi.org/10.1021/acs.langmuir.5b02198.

(6) Nagle, J. F.; Tristram-Nagle, S. Structure of Lipid Bilayers. *Biochim. Biophys. Acta BBA - Rev. Biomembr.* **2000**, *1469* (3), 159–195. https://doi.org/10.1016/S0304-4157(00)00016-2.
